# Cardiometabolic Biomarkers at Age 44-45 in the Psychosis Spectrum: The British National Child Development Study

**DOI:** 10.1093/schbul/sbag040

**Published:** 2026-05-05

**Authors:** Eugenia Kravariti, Abraham Reichenberg, Anna Maria Fragkaki, Robin M Murray, George B Ploubidis

**Affiliations:** Department of Psychosis Studies, Institute of Psychiatry, Psychology, and Neuroscience, King’s College London, London SE5 8AF, United Kingdom; Department of Psychosis Studies, Institute of Psychiatry, Psychology, and Neuroscience, King’s College London, London SE5 8AF, United Kingdom; Department of Psychiatry, Icahn School of Medicine at Mount Sinai, New York, NY 10029, United States; Department of Psychosis Studies, Institute of Psychiatry, Psychology, and Neuroscience, King’s College London, London SE5 8AF, United Kingdom; First Department of Psychiatry, School of Medicine, National and Kapodistrian University of Athens, Athens 115 28, Greece; Department of Psychosis Studies, Institute of Psychiatry, Psychology, and Neuroscience, King’s College London, London SE5 8AF, United Kingdom; Centre for Longitudinal Studies, Social Research Institute, University College London, London WC1H 0AL, United Kingdom

**Keywords:** cardiometabolic, birth cohort, longitudinal, psychosis spectrum, early life adversity

## Abstract

**Background:**

Excess mortality in psychotic disorders is largely due to preventable cardiometabolic morbidity. Efforts to evaluate the link between the psychosis spectrum and cardiometabolic health have been confounded by early-life adversity (ELA) and biased sampling. This population-based study examined prospective associations between psychosis-spectrum status and cardiometabolic biomarkers at age 44-45, adjusting for ELA.

**Study Design:**

We analyzed data from the 2002/03 biomedical sweep of the British National Child Development Study (*n* = 9377; age 44-45). Psychosis-spectrum status (exposure; *n* = 171) was defined using repeated screening across adulthood (ages 23 to 44-45), including self-reported diagnoses, antipsychotic medication use, or professional help-seeking for hallucinations. Cardiometabolic biomarkers at age 44-45 (outcomes) were compared between individuals on the psychosis spectrum and psychosis-free controls (comparator; *n* = 2448). Analyses were conducted using unimputed and multiply imputed datasets (*n* = 7391-9298), adjusting for 24 indicators of ELA.

**Study Results:**

In both unimputed/imputed analyses, individuals on the psychosis spectrum had significantly worse cardiometabolic profiles. Adjusted results showed elevated abdominal obesity (exp(b), 1.404; 95% CI, 1.177-1.676; *P* < .001), higher glycated hemoglobin (B = 0.321; 95% CI, 0.089-0.553; *P* = .008), lower high-density lipoprotein cholesterol (B = –4.472; 95% CI, –7.782 to –1.162; *P* = .009), and increased fibrinogen (B = 4.542; 95% CI, 0.939-8.144; *P* = .015) compared to controls.

**Conclusions:**

Overcoming early-life confounders and biases that limited prior research, our study demonstrates a robust, independent association between psychosis-spectrum status and cardiometabolic dysfunction at age 44-45. These findings underscore the urgent need for comprehensive screening, treatment, and monitoring of cardiometabolic morbidity in psychosis, guided by a life-course perspective.

## Introduction

People with psychotic disorders have a life expectancy 15-20 years shorter than that of the general population.^1^^,^^2^ This mortality gap persists—or even widens—over time,^3–6^ and 2 out of 3 deaths result from preventable physical disorders.^7^ Cardiovascular, other circulatory, and metabolic disorders are leading causes of mortality^3^^,^^5^ and among the most studied comorbidities in this clinical population.^8^

The etiology of cardiometabolic comorbidity in psychosis-spectrum disorders is complex and likely reflects the interplay of shared biological vulnerability^9–11^ and downstream behavioral and treatment-related factors that are highly prevalent among people with spectrum disorders.^7^^,^^8^^,^^12^ Genetic and neurobiological mechanisms implicated in psychosis have been linked to metabolic dysregulation and inflammatory processes,^9–11^^,^^13^ while individuals on the psychosis spectrum also show markedly higher rates of smoking, physical inactivity, poor diet, and exposure to antipsychotic medications with known metabolic side effects.^7^^,^^8^^,^^12^^,^^14–16^ These adult factors are strongly associated with obesity, insulin resistance, dyslipidemia, and cardiovascular disease in the general population^17–23^ and are therefore considered key mechanisms through which psychosis-spectrum status may contribute to elevated cardiometabolic risk across the life course.

An important upstream factor that may confound observed associations between the psychosis spectrum and cardiometabolic morbidity is early-life adversity (ELA), including perinatal events, lower socioeconomic status, abuse, neglect, and other adverse experiences. ELA is reported to increase the risk of both physical and mental health disorders, including psychosis,^24–29^ via mechanisms that are thought to include prolonged or dysregulated stress signaling, altered metabolism, mitochondrial dysfunction, inflammation, oxidative stress, telomere erosion, and biological aging.^28^ Consequently, to isolate the contribution of psychosis spectrum disorders and their modifiable correlates to cardiometabolic disorders in adult life, it is crucial to account for the pleiotropic effects of ELA. Unfortunately, this has been exceedingly challenging in existing research, which has mostly relied on health records and cross-sectional designs.^8^^,^^30^

We note 2 further limitations: First, primary, secondary, and tertiary health records only capture treatment-seeking individuals or those with complex care needs.^8^^,^^30^ As such, they are likely to miss non-help-seeking young adults with psychotic disorders in the community,^31^ those with complete recovery from a single episode of schizophrenia and no further episodes,^32^ and those who die from a physical condition that is diagnosed only after death—a risk which is significantly higher in people with than those without psychoses.^33^^,^^34^ Second, both mental and physical health are dimensional phenomena, but most studies^8^^,^^30^ have examined their inter-relationships using diagnostic categories. This analytic approach leads to a loss of statistical sensitivity and to an under-representation of the full spectrum of illness severity in the population.

The above limitations underscore a compelling imperative to understand the relationship between psychosis and cardiometabolic health from a general-population, life-course perspective. The 1958 National Child Development Study (NCDS)^35^ offers a unique context to address this aim, overcoming earlier methodological limitations. Using data from the 2002/03 biomedical sweep (age 44-45; *n* = 9377; population), we examined whether psychosis-spectrum status (exposure), screened for between ages 23 and 44-45, is prospectively associated with worse cardiometabolic biomarkers at age 44-45 (outcomes) compared with psychosis-free status (comparator), after adjusting for ELA indicators (covariates).

Previous evidence has indicated that adversity sampled from earlier developmental periods, assessed comprehensively, and including stress response measures alongside objective indices of adversity, shows greater and longer-lasting effects on physiological parameters.^28^ In line with this evidence, our analysis included a wide range of ELA indicators (24 variables) from the earliest developmental period available (0-7 years), including both objective markers of adversity (eg, socio-economic status), and cognitive, emotional and behavioral responses to life stressors (eg, internalizing and externalizing symptoms).

## Methods

### Population

Our study population comprised all NCDS participants of the 2002/03 biomedical sweep [*n* = 9377; 58.6% of eligible population (*n* = 16 003); age 44-45; 50.3% female]. Additional information on response rates and missingness is available from the Centre for Longitudinal Studies (CLS) website. The NCDS (also known as the 1958 British Birth Cohort Study) follows the lives of a nationally representative UK birth cohort, an initial 17 415 people born in England, Scotland, and Wales in a single week of 1958.^35^ Participants have been surveyed 12 times since birth, providing rich prospective data on social, biological, physical, and psychological phenotypes.

### Exposure

The exposure of interest was psychosis-spectrum status in adulthood, identified between ages 23 and 44-45. Participants were classified as being on the psychosis spectrum following a detailed review of the NCDS data, and using screening items for (1) “psychotic conditions” [codes F20-29 of the International Classification of Diseases (ICD): schizophrenia, schizotypal, delusional, and other non-mood psychotic disorders], (2) prescribed medications for “‘psychoses and related disorders’, and (3) auditory or visual hallucinations which had prompted professional help-seeking at any time-point between ages 8 and 44-45. The above information was self-reported by participants during any of the 1981 (age 23), 1991 (age 33), 2000 (age 42) and 2002/03 (age 44-45) data sweeps. Endorsing any number of the psychosis spectrum screening items led to classification under ‘psychosis spectrum’”.

In the unimputed dataset (we used multiple imputation for missing values; see *Statistical Modelling: Missing Data*), 244 participants screened positive for 1 or more screening items, after excluding 7 participants who reported an onset of psychosis-spectrum experiences before age 8. The latter were excluded to allow an examination of *prospective* associations between psychosis-spectrum status and cardiometabolic outcomes at age 44-45, controlling for ELA between birth and age 7. Of the 244 psychosis spectrum participants, 171 (70%) also participated in the biomedical survey (“psychosis-spectrum cases” or “cases”). The mean ± SD reported age of onset of psychosis-spectrum experiences was 29.34 ± 7.81 years (range 10-41 years). Of the 171 cases, 99 (57.89%) endorsed 1 screening item and 72 (42.11%) endorsed 2-3 screening items. The screening items and the number of cases ascertained in each wave are presented in Supplementary Table S1.

### Comparator

Of the biomedical survey population (*n* = 9377), 2448 participants (26.11%) both participated in the biomedical survey and provided complete and uniformly negative responses across all psychosis-screening items, forming the psychosis-free comparator group (“psychosis-free controls” or “controls”).

A flow chart of the derivation of the analytic cohort in the unimputed dataset is presented in Supplementary Figure S1.

### Sample Representativeness and Face Validity of Case Ascertainment

The full biomedical survey sample was broadly representative of the surviving cohort in 2002/03, and any bias was mostly present before the survey.^36^ The psychosis-spectrum cases (full sample: 244; biomedical survey sample: 171) represented 1.5% and 1.8% of the eligible (*n* = 16 003) and achieved (*n* = 9377) biomedical survey samples, respectively. Both estimates are proximal to the prevalence of schizophrenia-spectrum disorders in an earlier population study (1.44%).^37^ The mean ± SD age of onset of psychosis-spectrum difficulties in the NCDS was 29.34 ± 7.81 years (range 10-41), consistent with epidemiological estimates reported in a meta-analysis (typically 25-35 years).^38^ The epidemiological, neurobehavioral, and psychosocial profiles of the psychosis-spectrum cases reflected the atypical neurodevelopment known to characterize schizophrenia (significantly lower birth weight, lower cognitive ability at age 7, and higher externalizing symptoms at age 7 compared to population controls; see Results). These characteristics provided face validity to our definition of the “psychosis spectrum.”

### Primary Outcomes

The 2002/03 biomedical survey obtained objective measures of ill health and biomedical risk factors at age 44-45 via face-to-face administration (nurse visit) and via paper self-completion questionnaires.

Our primary outcomes were cardiometabolic biomarkers assessed at age 44-45, including fibrinogen and C-reactive protein (CRP)—both markers of inflammation and cardiovascular disease; glycated hemoglobin (HbA1c)—an index of glucose metabolism throughout the previous 30-90 days, and a marker of pre-diabetes and diabetes; high-density lipoprotein (HDL) cholesterol and low-density lipoprotein (LDL) cholesterol—markers of cardiovascular risk; and high blood pressure—recorded as present, if the mean value of all valid readings (maximum of 3) of systolic and diastolic blood pressure exceeded 140/90 mm Hg. Participants who reported using blood pressure-related medication were also classified as having high blood pressure. As a measure of abdominal obesity, waist and hip circumferences were measured, and waist-to-hip ratio was used (≥0.94 in male individuals; ≥0.85 in female individuals).^39–41^ With the exception of HbA1c, which represents the percentage of total hemoglobin, all continuous biomarkers were transformed with the natural logarithm and multiplied by 100, so all ordinary least squares regression coefficients can be interpreted as percentage differences in means.^42^ Appropriate corrections for medication use were also applied, as detailed in Ploubidis and colleagues.^27^ Details on the measurement procedures and equipment are available on the biomedical sweep technical report.^43^

In addition to the above main outcomes, we examined the prevalence of secondary outcomes (see *Other Outcomes* and Appendix 1 of the Supplementary Material) among psychosis-spectrum cases and controls.

### Other Outcomes

To facilitate comparisons with the existing literature, we further estimated the prevalence of prediabetes, diabetes, and metabolic syndrome among cases and controls (for a definition of metabolic syndrome, see Appendix 1 of the Supplementary Material). While mortality was not the primary focus of this study, we also examined all-cause mortality by the year 2016 (age 58) to enhance the interpretability of our findings and examine their alignment with relevant research. All secondary outcomes were derived from frequency counts using the unimputed dataset.

### Covariates

Models adjusted for ELA indicators from 0 to 7 years (24 covariates), encompassing both objective markers of adversity (eg, socio-economic status) and physiological, psychosocial, and behavioral responses to life stressors (eg, affective symptoms and conduct problems). The ELA covariates are detailed in Appendix 2 of the Supplementary Material, and included birth characteristics, measures of socio-economic position, parental and family characteristics, cognitive ability, emotional and behavioral adjustment, and physical health.

### Statistical Modeling: Missing Data

We addressed missing data using multiple imputation by chained equations,^44^ generating 25 imputed datasets (*n* = 7391-9298) to reduce bias and preserve statistical power. Each statistical model was estimated separately within all imputed datasets, and results were subsequently pooled using Rubin’s rules^45^^,^^46^ to obtain combined point estimates and robust standard errors that account for both within- and between-imputation variability. Further details are provided in Appendix 3 of the Supplementary Material.

### Statistical Modeling: Analytic Strategy

Supplementary Figure S2 illustrates the hypothesized relationships among the study variables. Using the imputed dataset and a series of multivariable models, we examined prospective associations between psychosis-spectrum status and cardiometabolic outcomes at age 44-45, while controlling for ELA. Psychosis-related adult factors (eg, smoking, physical activity, diet, and antipsychotic exposure) were not adjusted for, as they were conceptualized a priori as intermediate variables linking psychosis-spectrum status with cardiometabolic outcomes. For continuous outcomes (fibrinogen, CRP, HbA1c, HDL/LDL cholesterol), we applied ordinary least squares regression. Abdominal obesity and high blood pressure were analyzed as binary outcomes to mitigate model instability arising from potential non-linear associations (eg, both very low and very high blood pressure and/or waist-to-hip ratio may indicate poor health). For these binary outcomes, we used a log-binomial model with robust standard errors to estimate risk ratios, as both outcomes were common (>20%), helping to avoid bias from the non-collapsibility of the odds ratio.^47^ Analyses were conducted using Stata version 18 (StataCorp).

### Sensitivity and Subgroup Analyses

To examine potential heterogeneity and the internal validity of the psychosis spectrum classification, and to enhance transparency of reporting, we conducted a series of prespecified sensitivity and subgroup analyses. These included univariable case–control comparisons stratified by the number of screening items endorsed (1 vs 2-3 items), as well as comparisons between controls and cases endorsing 1 screening item, cases endorsing 2-3 screening items, cases endorsing hallucination-related screening items only, and case–control analyses excluding cases who endorsed only antipsychotic medication screening items or only psychotic condition screening items. In addition, to assess within-group heterogeneity, we examined whether earlier age of onset was associated with worse cardiometabolic outcomes among individuals on the psychosis spectrum.

### Reporting Strategy

All descriptive statistics and univariable comparisons between cases and controls, or within the psychosis-spectrum group, including sensitivity analyses, are based on the unimputed dataset (Tables 1 and 2, Supplementary Tables S1–S8). To reduce bias and maintain power in the multivariable models, all inferential statistics addressing the main research hypothesis are based on the imputed dataset (Table 3, Supplementary Tables S9–S15). The analysis of `other outcomes' is based on the unimputed dataset (Supplementary Table S16). This study is reported in accordance with the STROBE (Strengthening the Reporting of Observational Studies in Epidemiology) guidelines. A completed STROBE checklist is provided in Appendix 4 of the Supplementary Material.

**Table 1:** Cardiometabolic Biomarkers^a^ at Age 44-45 in Participants on the Psychosis Spectrum (*n* = 171) and Controls (*n* = 2448)

	Psychosis spectrum (*n* = 171)^b^		Control (*n* = 2448)			
Biomarker	Mean	SD	Mean	SD	t	*P*
	mg/dL or mg/L^c^					
						
Fibrinogen	314.93	68.52	294.19	60.98	−3.463	**<.001**
C-reactive protein	2.81	4.52	2.10	5.01	−2.618	**.004**
High-density lipoprotein cholesterol	57.15	16.24	60.94	15.41	3.035	**.001**
Low-density lipoprotein cholesterol	133.92	33.35	132.01	34.62	−0.804	.211
			
	DCCT-HbA1c^d^					
						
Glycated hemoglobin (HbA1c)	5.43	1.06	5.22	0.57	−3.507	**<.001**
						
	***n***	**%**	***n***	**%**	**Pearson χ** ^2^	***P***
						
Abdominal obesity	74	44.3	665	27.2	22.425	**<.001**
High blood pressure	34	20.4	378	15.6	2.605	.107
						
						

a Descriptive characteristics and univariable group comparisons are based on the unimputed dataset. Statistically significant group comparisons are indicated by *P* values <.05; for enhanced visibility, P values <.05 in the last column are shown in boldface.

b Of the 171 participants on the psychosis spectrum, 99 (57.9%) endorsed 1 screening item (one-item group), while 72 (42.1%) endorsed 2-3 items (2/3-item group). There were no statistically significant differences between subgroups across any cardiometabolic biomarkers, including fibrinogen (mean ± SD: 317.45 ± 72.55 vs 311.26 ± 62.72; *P* = .978), C-reactive protein (3.20 ± 5.42 vs 2.23 ± 2.67; *P* = .888), high-density lipoprotein cholesterol (58.34 ± 18.25 vs 55.28 ± 12.48; *P* = .384), low-density lipoprotein cholesterol (132.20 ± 33.97 vs 136.36 ± 32.64; *P* = .577), and glycated hemoglobin (HbA1c) (5.43 ± 1.24 vs 5.42 ± 0.74; *P* = .883). Similarly, the prevalence of abdominal obesity (45.9% vs 41.3%; *P* = .547) and high blood pressure (20.6% vs 18.6%; *P* = .744) did not differ between groups.

c Fibrinogen, high-density lipoprotein cholesterol and low-density lipoprotein cholesterol are expressed in mg/dL; C-reactive protein is expressed in mg/L.

d DCCT-HbA1c is expressed as % of overall hemoglobin and aligned to the assay used in the Diabetes Control and Complications Trial (DCCT).

**Table 2:** Indicators of Early-Life Adversity^a^ Among Participants on the Psychosis Spectrum (*n* = 171) and Controls (*n* = 2448)

	Psychosis spectrum		Control			Psychosis spectrum		Control	
Early-life characteristic	*n*	%	*n*	%	Early-life characteristic	*n*	%	*n*	%
Female	89	52	1309	53.5	Region at CM’s birth				
Mother not married at birth	8	5	64	2.7	North	13	8.1	164	7
**Breastfed** ^d^	86	58.9	1576	72.6	North West	23	14.4	285	12.2
Low birth weight	11	7.1	119	5.2	E. & W. Riding	13	8.1	204	8.7
**Mother smoked during pregnancy** ^b^	64	40.5	716	30.9	North Midlands	15	9.4	181	7.7
Mother employed up to CM’s age 5	52	36.4	620	29.6	Midlands	19	11.9	226	9.6
**Housing tenure – family owned** ^d^	43	29.7	1013	46.4	East	9	5.6	212	9.1
**Housing difficulties** ^c^	18	12.9	140	6.8	South East	26	16.3	492	21
**Financial difficulties** ^d^	22	16.7	111	5.7	South	5	3.1	136	5.8
Nocturnal enuresis, age 7	20	13.7	243	11.2	South West	9	5.6	126	5.4
**Parental divorce/separation by age 7** ^b^	11	7.9	68	3.3	Wales	8	5	123	5.3
**Separation from mother >1 month** ^b^	23	16.2	215	10.1	Scotland	20	12.5	193	8.2
Parents do not want child to stay at school	7	5.9	71	3.7		**Mean**	**SD**	**Mean**	**SD**
Mother hardly ever reads to child	20	13.9	315	14.6	Mother’s age at last birthday	27.16	5.5	27.75	5.6
Mother not interested in child’s education^c^	28	21.1	251	12.2	**Cognitive ability summary, age 7** ^d^	−0.19	0.98	0.16	0.76
Father’s social class at birth					**Externalizing symptoms** ^b^	0.16	1.13	−0.05	0.95
I	4	2.7	123	5.5	Internalizing symptoms	0.02	0.29	0.01	0.28
II	16	10.9	338	15.1	**Medical examination summary, age 7** ^b^	9.67	2.41	9.25	2.12
III	93	63.3	1366	61.1	Body Mass Index (BMI) at age 7	16.02	1.73	15.84	1.59
IV	21	14.3	249	11.1	Number of household amenities	3.94	0.811	4.02	0.71
V	13	8.8	158	7.1					

Abbreviation: CM: Cohort Member

a Using the unimputed dataset and univariable group comparisons.

b ***P* ≤ .05.**

c ***P* < .01.**

d ***P* ≤ .001.**

**Table 3:** Associations of Psychosis Spectrum with Cardiometabolic Biomarkers at Age 44-45,^a^ Adjusting for 24 Indicators of Early-Life Adversity^b^

	*n*	Coefficient^c^/exp(b)^d^	SE	t	*P < =*	95% CI	
**Continuous biomarkers**							
Glycated hemoglobin (HbA1c)	7923	0.32	0.11	2.84	**0.008**	0.09	0.55
High-density lipoprotein (HDL) cholesterol	7808	−4.47	1.65	−2.71	**0.009**	−7.78	−1.16
Low-density lipoprotein (LDL) cholesterol	7391	2.99	2.93	1.02	0.32	−2.97	8.96
Fibrinogen	7670	4.54	1.78	2.55	**0.02**	0.94	8.14
C-reactive protein (CRP)	7466	12.11	9.11	1.33	0.19	−6.27	30.49
**Binary biomarkers**							
Abdominal obesity	9298	1.40	0.12	3.88	**0.001**	1.18	1.68
High blood pressure	9229	1.20	0.20	1.09	0.28	0.86	1.69

a Using the imputed dataset (*n* = 7391-9298) and multivariable analyses.

b Early-life (0-7 years) socio-economic, birth, parental, family, cognitive, emotional, behavioral and physical health factors including sex, nocturnal enuresis at age 7, mother’s age at last birthday, cohort member ever breastfed, low birth weight, mother smoked during pregnancy, mother employed up to cohort member’s age 5, housing tenure, housing difficulties, financial difficulties, divorce/separation by cohort member’s age 7, cognitive ability at age 7, externalizing symptoms, internalizing symptoms, summary of medical examinations at age 7, maternal separation for over 1 month, parents want child to stay at school, mother hardly ever reads to child, mother not interested in child’s education, number of household amenities, mother not married at birth, father’s social class at birth, region at cohort member’s birth, and Body Mass Index (BMI) at age 7.

c Continuous biomarkers. With the exception of glycated hemoglobin, which represents the percentage of total hemoglobin, all continuous biomarkers were transformed with the natural logarithm and multiplied by 100, so all ordinary least squares regression coefficients can be interpreted as percentage differences in means.

d Binary biomarkers.

### Ethics and Consent

All procedures contributing to this work comply with the ethical standards of the relevant national and institutional committees on human experimentation and with the Helsinki Declaration of 1975, as revised in 2013. All procedures involving human subjects were approved by the University College London Institute of Education Research Ethics Committee. The NCDS biomedical survey was approved by the South-East Multicenter Research Ethics Committee (MREC reference 01/1/44). Written informed consent for all measures and samples was obtained from all participants during the interview. More information on the NCDS Ethical approval procedures 1958-2013 can be found on the CLS website.

## Results

### Cardiometabolic and Early-Life Characteristics

The cardiometabolic and early-life (0-7 years) characteristics of cases and controls, and the results of the univariable comparisons between the 2 groups, are presented in Tables 1 and 2 (unimputed dataset). The early-life characteristics of the eligible biomedical survey sample are presented in Supplementary Table S2 (unimputed dataset). Compared to controls, psychosis-spectrum cases showed more adverse cardiometabolic and early-life profiles (Tables 1 and 2). Of the extended pool of psychosis-spectrum cases (*n* = 244), those who did not take part in the biomedical survey (*n* = 73) showed lower cognitive ability and poorer physical health in early life compared to those who participated (*n* = 171) (Supplementary Table S3).

### Prospective Associations of Psychosis Spectrum with Cardiometabolic Outcomes at Age 44-45, Adjusting for ELA

Table 3 presents a summary of the statistical associations between psychosis-spectrum status and cardiometabolic outcomes at age 44-45 after multivariable adjustment for ELA (imputed dataset). The full details of the multivariable models are presented in Supplementary Tables S9–S15. Graphical summaries of the statistical associations for the continuous and binary cardiometabolic outcomes are presented in Supplementary Figures S3 and S4. All ordinary least squares regression coefficients (continuous outcomes) can be interpreted as percentage differences in means between the psychosis-spectrum cases and controls.^42^

Statistically significant associations emerged for fibrinogen (*P* = .015), HDL cholesterol (*P* = .009), HbA1c (*P* = .008) and abdominal obesity (*P* < .001) (Table 3 and Supplementary Tables S9, S10, S12, and  S14). The relative increase in the odds of obesity among cases was 40.4%. Non-significant statistical associations emerged for CRP, LDL cholesterol, and high blood pressure (Table 3, Supplementary Tables S11, S13, and  S15). These findings mirror the results of the univariable group comparisons in the unimputed dataset (Table 1; also see *Sensitivity and Subgroup Analyses* below) except for CRP, which showed a significant association with the psychosis spectrum before (Table 1; also see *Sensitivity and Subgroup Analyses* below), but not after multiple imputation and multivariable adjustment for ELA (Table 3, Supplementary Table S13). Of the latter covariates, male sex, lower cognitive ability at age 7, and higher Body Mass Index (BMI) at age 7 showed the most consistent significant associations with cardiometabolic outcomes at age 44-45 (Supplementary Tables S9–S15).

### Other Outcomes

Among psychosis-spectrum cases, the prevalence of prediabetes, diabetes, and metabolic syndrome was 13%, 3%, and 25%, respectively, compared to 7%, 2%, and 17% among controls (Supplementary Table S16; unimputed dataset). Of the 2448 controls, 61 (2.49%) had died by the year 2016 (age 58). The corresponding data for all identified psychosis-spectrum cases (*n* = 244) and those participating in the biomedical survey (*n* = 171) were 29 (11.89%) and 12 (7.02%), respectively. These data point to a 3- to 5-fold increase in all-cause mortality among psychosis-spectrum cases relative to controls.

### Sensitivity and Subgroup Analyses

There were no statistically significant differences in any cardiometabolic biomarkers between cases who endorsed 1 screening item (*n* = 99) and those who endorsed 2-3 screening items (*n* = 72) (Table 1, footnote b).

The results of the sensitivity analyses are presented in Supplementary Tables S4–S8. Across all sensitivity analyses, the direction of associations and the pattern of statistical significance remained broadly consistent with the main findings, supporting the robustness of the psychosis-spectrum classification. Earlier age of onset was associated with worse HDL cholesterol levels among the cases (coefficient = 0.921; 95% CI, 0.206-1.637; *P* = .012), with no significant associations observed for other cardiometabolic outcomes.

## Discussion

Drawing on a rare birth cohort, followed prospectively for the past 68 years, our study offers unique insights into the cardiometabolic profile of adults with lived experiences of the psychosis spectrum in the general population. Our findings highlight high rates of abdominal obesity, along with abnormalities in HDL cholesterol, HbA1c, and fibrinogen levels at age 44-45—critically, after controlling for ELA. The latter is a major public health concern with long-lasting physiological consequences. Being associated with risk for both mental and physical disease,^28^ ELA is likely to have obscured the net influence of psychosis-spectrum disorders and their modifiable correlates on cardiometabolic outcomes in earlier studies.

The degree of biomarker dysregulation among psychosis-spectrum cases fell between the levels reported in the literature for patients with early- vs multi-episode psychosis. The prevalence of abdominal obesity (44%) and metabolic syndrome (25%) markedly exceeded the ranges reported for early psychosis in 4 meta-analyses (obesity: 17%-27%; metabolic syndrome: 10%-13%),^48–51^ approaching the levels reported for chronic schizophrenia patients in their fifth decade of life (obesity: 50%-53%; metabolic syndrome: 33%-35%).^48^^,^^49^^,^^51^ In contrast, the prevalence of prediabetes (13%) and diabetes (3%) fell just above or within the ranges reported for early psychosis in the same meta-analyses (prediabetes: 64-9%; diabetes: 1%-9%),^49–51^ suggesting less advanced pathology.

Our findings indicate that the cardiometabolic abnormalities of middle-aged adults on the psychosis spectrum in the general population largely overlap with those of help-seeking and complex-care psychosis patients who are ascertained through primary, secondary, and tertiary health records.^8^^,^^48–51^ This suggests a gradient of cardiometabolic risk operating on the entire psychosis continuum. This interpretation further agrees with reports of higher rates of cardiometabolic risk factors among individuals at ultra-high risk for psychosis.^52^ Importantly, based on universal epidemiological estimates^1^^,^^2^ and on our finding of a 3- to 5-fold increase in all-cause mortality by age 58, many psychosis-spectrum cases in the NCDS (currently in their late 60s) are likely now approaching or have already reached their life expectancy. Strikingly, this evidence suggests a missed 20-year window of opportunity to address key risk factors for premature mortality among people on the psychosis spectrum in the general population, including modifiable factors such as smoking, excessive alcohol use, physical inactivity, poor diet, adverse effects of psychiatric medications, stigma, limited healthcare access, inadequate health monitoring, and poor treatment adherence.^7^^,^^8^^,^^12^ Additionally, potential shared genetic underpinnings with schizophrenia may also play a role.^9–11^

Our finding of a 40.4% increase in the odds of obesity among psychosis-spectrum cases, following correction for ELA, has significant implications. Obesity shows excessive prevalence among individuals with schizophrenia,^8^^,^^53–55^ contrary to reports of reduced genetic risk of obesity in this population,^10^ and against evidence that obesity is more likely to follow than precede a schizophrenia diagnosis.^56^ This evidence underscores the likely dominant role of modifiable risk factors in obesity in the context of psychosis. As obesity is an established mediator of type 2 diabetes and cardiovascular disorders,^57^ excessive abdominal fat accumulation is a likely pivotal stage in the physiological cascade that reduces life expectancy among individuals with psychoses. It is noteworthy that obesity is one of the most distressing side effects of medication reported to mental health helplines,^58^ acting as a multi-faceted barrier to social engagement, treatment adherence, and sustained recovery.^8^ In addition, a combination of obesity and schizophrenia shows accentuated brain alterations compared to either phenotype in isolation.^59^ Taken together, these findings emphasize that treating obesity is a paramount therapeutic imperative for individuals on the psychosis spectrum,^60^ urging for lifestyle interventions^61^ and for treatment algorithms informed by the highly variable profiles of metabolic side effects of different antipsychotics.^62^

Although not a primary focus of the present study, both lower cognitive ability and higher BMI at age 7 showed statistically significant associations with cardiometabolic health at age 44-45 in most multivariable models. However, only cognitive ability at age 7 was significantly poorer among future psychosis-spectrum cases compared to controls. Thus, both childhood factors seem to be related to adult physical health, but cognitive impairment is considered integral to the schizophrenia diathesis,^63–65^ while increased BMI is a likely by-product of treated psychosis.^10^^,^^56^ Our findings support recommendations for the concurrent treatment of cognitive deficits and obesity in schizophrenia.^66^^,^^67^

### Strengths and Limitations

Our analysis provides a crucial window into the cardiometabolic profile of a birth cohort as it presented 2 decades ago. Despite this time lag, our findings remain highly relevant today. Indeed, alarming recent evidence suggests worsening physical health across successive cohorts in Europe and the United States, particularly among those born since 1945,^68^ alongside a persistent—or even widening—mortality gap between individuals with and without psychoses.^3–6^

Epidemiological samples are less biased than clinically ascertained samples with psychosis. An important novelty of our study is its focus on a continuum of objective markers of cardiometabolic health in relation to a continuum of psychosis, both continua intersecting a population-based sample. Critically, the life-course framework of NCDS enabled a crucial separation of ELA from psychosis-related contributions to adult cardiometabolic health.^24^^,^^69^ Another important novelty is the blanket, inclusive, and accessible investigative approach of the NCDS, which created a level playing field for the study of cardiometabolic biomarkers among psychosis-spectrum and control populations. The uniformity and laboratory precision of this approach diminished the confounding influences of diagnostic overshadowing, differential patterns of help-seeking and differential pathways to physical health diagnoses^7^ between the 2 populations.

Our study addressed a methodological and evidential gap between research findings arising from administrative health records and those drawn from and contextualized within epidemiological settings. The 2 approaches are powerfully complementary, but the latter is uniquely placed to inform public health interventions and to tap into some of the “missing morbidity” of psychosis (eg, young adults with psychotic disorders who have not yet received a diagnosis)—whilst also accessing the more visible segments of the psychosis spectrum (eg, individuals receiving health care in the community).

Our study aimed to separate ELA from psychosis-related contributions to cardiometabolic health at age 44-45, with a primary emphasis on psychosis-related factors. Isolating the impact of individual psychosis-related exposures (eg, antipsychotic medication) or specific ELA indicators (eg, physiological vs sociodemographic stressors in childhood) was beyond the scope of our hypothesis-testing study. An examination of potential causal effects of ELA on cardiometabolic outcomes at age 44-45, with appropriate adjustment for psychosis-related factors, would require early-life–focused analyses, representing an important direction for future research.

Our study relied on screening items rather than diagnostic interviews for the definition of the “psychosis spectrum.” This strategy reduced diagnostic resolution and may have increased recall error, but, importantly, its accessibility is likely to have increased the representation of more vulnerable groups in our study. Equally, the biomedical survey may have missed cohort members from the severe end of the psychosis continuum. However, a higher representation of this segment of the clinical population would have likely accentuated an already adverse profile of cardiometabolic health.

Our study findings need to be viewed in the context of some limitations: We did not stratify by or adjust for psychiatric comorbidity, as restricting our analyses to participants with sufficient data on both psychosis-spectrum status and psychiatric comorbidity would substantially reduce the case sample and introduce selection bias; moreover, psychiatric comorbidity may plausibly lie on the causal pathway between psychosis-spectrum experiences and later cardiometabolic dysfunction (eg, via chronic stress), such that adjustment could constitute over-adjustment.

Although blood pressure and adiposity exist on a continuum, we used binary classifications to mitigate model instability arising from potential non-linear associations (eg, both very low and very high blood pressure and/or waist-to-hip ratio may indicate poor health), which may have obscured more nuanced cardiometabolic risk gradients. Certain ELA effects (eg, abuse) did not feature in our statistical models, but were likely captured by variables reflecting cognitive, emotional, and behavioral responses to a broad range of stressors. Most (~98%) of the NCDS cohort members are of White British ethnicity,^70^ reflecting the demographic composition of the United Kingdom in the late 1950s. This relative lack of ethnic diversity limited our ability to examine ethnicity-related differences and constrains the generalizability of our findings (eg, to more ethnically diverse contemporary populations). Addressing this uncertainty represents an important direction for future research. Finally, as this was an observational cohort study, the findings reflect associations rather than definitive causal relationships. Residual confounding cannot be fully excluded, and misclassification of psychosis-spectrum status and cardiometabolic outcomes is possible despite prospective data collection and detailed measurement.

In conclusion, our findings demonstrate a robust association between psychosis-spectrum status and cardiometabolic dysfunction at age 44-45 in the general population, independent of ELA. The marked increase in the odds of obesity, and the likely interplay of early- and adult-life influences on cardiometabolic outcomes among people on the psychosis spectrum, suggest a complex relationship and highlight obesity as a high therapeutic imperative. Our findings underscore the need for sustained cardiometabolic screening, treatment, and monitoring in psychosis within a life-course framework.

## Supplementary Material

SZBLTN_-_ART_-_25_-_0622_Supplement_-_R_sbag040
