# Supplementary material for: Cardiometabolic Biomarkers at Age 44-45 in the Psychosis Spectrum: The British National Child Development Study
Source: Schizophr Bull. 2026 May 5;52(3):sbag040. doi: 10.1093/schbul/sbag040 (PMC13140664; doi:10.1093/schbul/sbag040)
Supplement: SZBLTN_-_ART_-_25_-_0622_Supplement_-_R_sbag040 [file szbltn_-_art_-_25_-_0622_supplement_-_r_sbag040.docx]

**Supplementary Material**

| **Table S1** Screening items for the psychosis spectrum and number of cases identified in each sweep (unimputed dataset) | Page 2 |
| --- | --- |
| **Table S2** Indicators of early-life adversity in the eligible 2002/3 biomedical survey sample (N=16003) (unimputed dataset) | Page 3 |
| **Table S3** Indicators of early-life adversity in the psychosis-spectrum cases who participated in the 2002/3 biomedical survey (n=171)  compared to those who did not participate (n=73) (unimputed dataset) | Page 4 |
| **Table S4** Sensitivity Analysis: Univariable comparisons in cardiometabolic biomarkers at age 44-45 between cases endorsing one screening item (N = 99) and controls (N = 2448) | Page 5 |
| **Table S5** Sensitivity Analysis: Univariable comparisons in cardiometabolic biomarkers at age 44-45 between cases endorsing two-to-three screening items (N = 72) and controls (N = 2448) | Page 6 |
| **Table S6** Sensitivity Analysis: Univariable comparisons in cardiometabolic biomarkers at age 44-45 between cases endorsing only hallucination-related screening items (N = 122) and controls (N = 2448) | Page 7 |
| **Table S7** Sensitivity Analysis: Univariable case-control comparisons in cardiometabolic biomarkers at age 44-45 excluding cases endorsing only antipsychotic medication-related screening items (N = 26 excluded) | Page 8 |
| **Table S8** Sensitivity Analysis: Univariable case-control comparisons in cardiometabolic biomarkers at age 44-45 excluding cases endorsing only psychotic condition-related screening items (N = 6 excluded) | Page 9 |
| **Table S9** Association of psychosis spectrum with glycated haemoglobin at age 44-45 (imputed dataset, N=7923) | Page 10 |
| **Table S10** Association of psychosis spectrum with high-density lipoprotein (HDL) cholesterol at age 44-45 (imputed dataset, N=7808) | Page 11 |
| **Table S11** Association of psychosis spectrum with low-density lipoprotein (LDL) cholesterol at age 44-45 (imputed dataset, N=7391) | Page 12 |
| **Table S12** Association of psychosis spectrum with fibrinogen at age 44-45 (imputed dataset, N=7670) | Page 13 |
| **Table S13** Associations of psychosis spectrum with C-reactive protein (CRP) at age 44-45 (imputed dataset, N=7466) | Page 14 |
| **Table S14** Association of psychosis spectrum with abdominal obesity at age 44-45 (imputed dataset, N=9298) | Page 15 |
| **Table S15** Association of psychosis spectrum with high blood pressure at age 44-45 (imputed dataset, N=9229) | Page 16 |
| **Table S16** Prevalence of pre-diabetes, diabetes and metabolic syndrome in the psychosis spectrum and control participants of the 2002/3 biomedical survey (unimputed dataset) | Page 17 |
| **Figure S1** Analytic cohort derivation (unimputed dataset) | Page 18 |
| **Figure S2** Directed acyclic graph illustrating the hypothesised prospective association^1^ between Psychosis-Spectrum Status and Cardiometabolic Outcomes at Age 44-45 | Page 19 |
| **Figure S3** Associations of Psychosis Spectrum with Continuous Cardiometabolic Biomarkers at Age 44-45 (Percentage Differences in Means between Cases and Controls) | Page 20 |
| **Figure S4** Associations of Psychosis Spectrum with Binary Cardiometabolic Outcomes at Age 44-45 (Odds Ratios) | Page 20 |
| **Appendix 1** Metabolic Syndrome | Page 21 |
| **Appendix 2** Indicators of early-life adversity (ELA) (covariates in multivariable models) | Page 22 |
| **Appendix 3** Missing Data | Pages 23-24 |
| **Appendix 4** STROBE Statement—checklist of items that should be included in reports of observational studies | Pages 25-27 |
| **References** | Page 28-29 |

**Table S1** Screening items for the psychosis spectrum and number of cases identified in each sweep (unimputed dataset)

| **Data sweep (age)** | **Number of cases identified ^a^** | **Variable name** | **Variable label** | **Value(s) used** |
| --- | --- | --- | --- | --- |
| 1981 (23 years) | 14 | psycat3 | 4D Psychotic conditions | 1=PRESENT |
| 1991 (33 years) | 109 | n504066 | CMI:79 F26a) Hearing/seeing things: Seen GP/Specialist (since Mar’81) | 6= Hear/seeing things |
|  | 73 | n504069 | CMI:79 F26c) Hearing/seeing things: Still a problem | <=2= Yes, most of the time/occasionally |
| 2000 (42 years) | 76 | mhprobs1 - mhprobs9 | MC:Seen specialist since refdate for mental disorder | 6= Hearing/seeing things no-one else does |
|  | 53 | mhstill6 | Still have problem hearing/seeing things | <=2= Yes, most of the time/occasionally |
|  | 28 | mhspec6 | Seen specialist/been to hosp re:hearing/seeing things | 1= Yes |
| 2002/3 (44-45 years) | 42 | drc1_m, drc2_m, drc3_m | (Modify from DRC1-3) Drug 1-3 BNF subchapter | 38= 0402 Psychoses&Rel.Disorders |
|  | 42 | medbia, medbia2, medbia3 | Drug 1-3 Used in the last seven days | 1= Yes |

^a^ The total number of cohort members who endorsed any of the screening items across sweeps was 244. Of these, 171 participated in the biomedical survey at age 44-45.

**Table S2** Indicators of early-life adversity in the eligible 2002/3 biomedical survey sample (N=16003) (unimputed dataset)

| **Early-life characteristic** | **N (%)** | **Total N** | **Early-life characteristic** | **N (%)** | **Total N** |
| --- | --- | --- | --- | --- | --- |
| Female | 7855 (42.3) | 16003 | Region at CM’s birth |  | 15000 |
| Mother not married at birth | 560 (3) | 14989 | *North* | 1081 (5.8) |  |
| Ever breastfed | 9087 (49) | 13331 | *North West* | 1937 (10.4) |  |
| Low birth weight | 862 (4.6) | 14501 | *E. & W. Riding* | 1267 (6.8) |  |
| Smoking during pregnancy | 4958 (26.7) | 14807 | *North Midlands* | 1126 (6.1) |  |
| Maternal employment up to age 5 | 3787 (20.4) | 12845 | *Midlands* | 1443 (7.8) |  |
| Housing tenure - owner | 5575 (30) | 13392 | *East* | 1069 (5.8) |  |
| Housing difficulties | 997 (5.4) | 12713 | *South East* | 2929 (5.8) |  |
| Financial difficulties | 1012 (5.5) | 11999 | *South* | 831 (4.5) |  |
| Nocturnal enuresis at age 7 | 1698 (9.1) | 13374 | *South West* | 849 (4.6) |  |
| Parental divorce/separation by age 7 | 529 (2.9) | 12819 | *Wales* | 806 (4.3) |  |
| Mother separation > 1 month | 1565 (8.4) | 13078 | *Scotland* | 1662 (9) |  |
| Parents want child to stay at school | 595 (3.2) | 11608 |  | **Mean (SD)** | **Total** |
| Mother hardly ever reads to child | 2134 (11.5) | 13281 | Mother's age at last birthday | 27.5 (5.72) | 14987 |
| Mother not interested in child’s education | 2077 (11.2) | 12783 | Cognitive ability summary, age 7 | -0.0073 (0.85) | 13257 |
| Father’s social class at birth |  | 14210 | Externalising symptoms | -0.0028 (0.999) | 13435 |
| *I* | 629 (3.4) |  | Internalising symptoms | 0.0004 (0.282) | 13435 |
| *II* | 1852 (10) |  | Medical examination summary, age 7 | 9.38 (2.29) | 13583 |
| *III* | 8568 (46.2) |  | Body mass index (BMI) at age 7 | 15.91 (1.77) | 12232 |
| *IV* | 1758 (9.5) |  | Number of household amenities | 3.96 (0.780) | 12594 |
| *V* | 1403 (7.6) |  |  |  |  |

CM: Cohort Member

**Table S3** Indicators of early-life adversity in psychosis-spectrum cases who participated in the 2002/3 biomedical survey (N=171) compared to those who did not participate (N=73) (unimputed dataset)

|  | **Participated (N=171)** | **Did not participate (N=73)** |  | **Participated (N=171)** | **Did not participate (N=73)** |
| --- | --- | --- | --- | --- | --- |
| **Early-life characteristic** | **N (%)** | **N (%)** | **Early-life characteristic** | **N (%)** | **N (%)** |
| Female | 89 (52) | 37 (56.1) | Region at CM’s birth |  |  |
| Mother not married at birth | 8 (5) | 1 (1.6) | *North* | 13 (8.1) | 5 (8.2) |
| Ever breastfed | 86 (58.9) | 33 (60) | *North West* | 23 (14.4) | 6 (9.8) |
| Low Birth Weight* | 11 (7.1) | 10 (17.5) | *E. & W. Riding* | 13 (8.1) | 15 (24.6) |
| Mother smoked during pregnancy | 64 (40.5) | 28 (46.7) | *North Midlands* | 15 (9.4) | 2 (3.3) |
| Mother employed up to CM’s age 5* | 52 (36.4) | 10 (19.2) | *Midlands* | 19 (11.9) | 5 (8.2) |
| Housing Tenure- Owner | 43 (29.7) | 18 (33.3) | *East* | 9 (5.6) | 2 (3.3) |
| Housing Difficulties | 18 (12.9) | 4 (7.5) | *South East* | 26 (16.3) | 9 (14.8) |
| Financial Difficulties | 22 (16.7) | 9 (18.4) | *South* | 5 (3.1) | 4 (6.6) |
| Nocturnal Enuresis at 7 | 20 (13.7) | 13 (23.6) | *South West* | 9 (5.6) | 3 (4.9) |
| Divorce/ separation by CM’s age 7 | 11 (7.9) | 4 (7.5) | *Wales* | 8 (5) | 3 (4.9) |
| Mother separation > 1 month | 23 (16.2) | 9 (17) | *Scotland* | 20 (12.5) | 7 (11.5) |
| Parents want child to stay at school | 7 (5.9) | 4 (9.3) |  | **Mean (SD)** | **Mean (SD)** |
| Mother hardly ever reads to child | 20 (13.9) | 8 (15.4) | Mother's age last birthday (yrs) | 27.16 (5.5) | 27.85 (6.8) |
| No mother’s interest in child’s education | 28 (21.1) | 15 (28.8) | Cognitive Ability Summary at 7** | -0.19 (0.98) | -0.63 (1.06) |
| Father’s social class at birth |  |  | Externalising symptoms | 0.16 (1.13) | 0.32 (1.15) |
| *I* | 4 (2.7) | - | Internalising symptoms | 0.02 (0.29) | 0.03 (0.29) |
| *II* | 16 (10.9) | 4 (6.7) | Summary of Medical Examinations at 7* | 9.67 (2.41) | 10.51 (2.73) |
| *III* | 93 (63.3) | 44 (73.3) | BMI at 7 | 16.02 (1.73) | 16.14 (1.77) |
| *IV* | 21 (14.3) | 7 (11.7) | Number of household amenities | 3.94 (0.81) | 4 (0.7) |
| *V* | 13 (8.8) | 5 (8.3) |  |  |  |

CM: Cohort Member

* p ≤ 0.05, ** p < 0.01, ***p ≤ 0.001

**Table S4** Sensitivity Analysis: Univariable comparisons in cardiometabolic biomarkers^1^ at age 44-45 between cases endorsing one screening item (N = 99) and controls (N = 2448)

|  | **Psychosis Spectrum (N=99)** | | **Control**  **(N=2448)** | |  |  |
| --- | --- | --- | --- | --- | --- | --- |
|  |  | | | |  |  |
|  |  | | | |  |  |
| **Biomarker** | **mean** | **s.d.** | **mean** | **s.d.** | **t** | ***P*** |
|  | mg/dL or mg/L ^2^ | | | |  |  |
|  |  |  |  |  |  |  |
| Fibrinogen | 317.45 | 72.55 | 294.19 | 60.98 | -2.929 | **0.002** |
| C-reactive protein | 3.20 | 5.42 | 2.10 | 5.01 | -2.313 | **0.010** |
| High-density lipoprotein cholesterol | 58.35 | 18.24 | 60.94 | 15.41 | 1.917 | **0.028** |
| Low-density lipoprotein cholesterol | 132.17 | 33.95 | 132.01 | 34.62 | -0.403 | 0.343 |
|  |  | | | |  |  |
|  | DCCT-HbA1c ^3^ | | | |  |  |
|  |  |  |  |  |  |  |
| Glycated Haemoglobin (HbA1c) | 5.43 | 1.24 | 5.22 | 0.57 | -2.888 | **0.002** |
|  |  |  |  |  |  |  |
|  | **N** | **%** | **N** | **%** | **Pearson χ^2^** | ***P*** |
|  |  |  |  |  |  |  |
| Abdominal obesity | 45 | 47.4 | 665 | 27.2 | 18.373 | **<.001** |
| High blood pressure | 21 | 21.2 | 378 | 15.6 | 2.219 | 0.136 |
|  |  |  |  |  |  |  |
|  |  |  |  |  |  |  |

^1^ Descriptive characteristics and univariable group comparisons are based on the unimputed dataset.

^2^ Fibrinogen, high-density lipoprotein cholesterol and low-density lipoprotein cholesterol are expressed in mg/dL; c-reactive protein is expressed in mg/L.

^3^ DCCT-HbA1c is expressed as % of overall haemoglobin and aligned to the assay used in the Diabetes Control and Complications Trial (DCCT).

**Table S5** Sensitivity Analysis: Univariable comparisons in cardiometabolic biomarkers^1^ at age 44-45 between cases endorsing two-to-three screening items (N = 72) and controls (N = 2448)

|  | **Psychosis Spectrum (N=72)** | | **Control**  **(N=2448)** | |  |  |
| --- | --- | --- | --- | --- | --- | --- |
|  |  | | | |  |  |
|  |  | | | |  |  |
| **Biomarker** | **Mean** | **s.d.** | **mean** | **s.d.** | **t** | ***P*** |
|  | mg/dL or mg/L ^2^ | | | |  |  |
|  |  |  |  |  |  |  |
| Fibrinogen | 314.72 | 62.86 | 294.19 | 60.98 | -1.977 | **0.024** |
| C-reactive protein | 2.31 | 2.62 | 2.10 | 5.01 | -1.371 | 0.085 |
| High-density lipoprotein cholesterol | 55.16 | 12.27 | 60.94 | 15.41 | 2.540 | **0.006** |
| Low-density lipoprotein cholesterol | 136.71 | 32.41 | 132.01 | 34.62 | -0.789 | 0.215 |
|  |  | | | |  |  |
|  | DCCT-HbA1c ^3^ | | | |  |  |
|  |  |  |  |  |  |  |
| Glycated Haemoglobin (HbA1c) | 5.41 | 0.72 | 5.22 | 0.57 | -2.231 | **0.013** |
|  |  |  |  |  |  |  |
|  | **N** | **%** | **N** | **%** | **Pearson χ^2^** | ***P*** |
|  |  |  |  |  |  |  |
| Abdominal obesity | 29 | 40.3 | 665 | 27.2 | 5.944 | **0.015** |
| High blood pressure | 13 | 19.1 | 378 | 15.6 | 0.606 | 0.436 |
|  |  |  |  |  |  |  |
|  |  |  |  |  |  |  |

^1^ Descriptive characteristics and univariable group comparisons are based on the unimputed dataset.

^2^ Fibrinogen, high-density lipoprotein cholesterol and low-density lipoprotein cholesterol are expressed in mg/dL; c-reactive protein is expressed in mg/L.

^3^ DCCT-HbA1c is expressed as % of overall haemoglobin and aligned to the assay used in the Diabetes Control and Complications Trial (DCCT).

**Table S6** Sensitivity Analysis: Univariable comparisons in cardiometabolic biomarkers^1^ at age 44-45 between cases endorsing only hallucination-related screening items (N = 122)^2^ and controls (N = 2448)

|  | **Psychosis Spectrum (N=122)** | | **Control**  **(N=2448)** | |  |  |
| --- | --- | --- | --- | --- | --- | --- |
|  |  | | | |  |  |
|  |  | | | |  |  |
| **Biomarker** | **mean** | **s.d.** | **mean** | **s.d.** | **t** | ***P*** |
|  | mg/dL or mg/L ^3^ | | | |  |  |
|  |  |  |  |  |  |  |
| Fibrinogen | 313.30 | 65.83 | 294.19 | 60.98 | -2.778 | **0.003** |
| C-reactive protein | 2.82 | 4.79 | 2.10 | 5.01 | -2.213 | **0.013** |
| High-density lipoprotein cholesterol | 56.92 | 14.64 | 60.94 | 15.41 | 2.554 | **0.005** |
| Low-density lipoprotein cholesterol | 133.91 | 34.44 | 132.01 | 34.62 | -0.750 | 0.227 |
|  |  | | | |  |  |
|  | DCCT-HbA1c ^4^ | | | |  |  |
|  |  |  |  |  |  |  |
| Glycated Haemoglobin (HbA1c) | 5.50 | 1.23 | 5.22 | 0.57 | -4.108 | **<.001** |
|  |  |  |  |  |  |  |
|  | **N** | **%** | **N** | **%** | **Pearson χ^2^** | ***P*** |
|  |  |  |  |  |  |  |
| Abdominal obesity | 51 | 42.9 | 665 | 27.2 | 13.732 | **<.001** |
| High blood pressure | 13 | 11.0 | 378 | 15.6 | 0.075 | 0.784 |
|  |  |  |  |  |  |  |
|  |  |  |  |  |  |  |

^1^ Descriptive characteristics and univariable group comparisons are based on the unimputed dataset.

^2^ Of the 122 participants who endorsed only hallucination-screening items, 62 endorsed a single hallucination-screening item item and 60 endorsed 2-3 hallucination-screening items.

^3^ Fibrinogen, high-density lipoprotein cholesterol and low-density lipoprotein cholesterol are expressed in mg/dL; c-reactive protein is expressed in mg/L.

^4^ DCCT-HbA1c is expressed as % of overall haemoglobin and aligned to the assay used in the Diabetes Control and Complications Trial (DCCT).

**Table S7** Sensitivity Analysis: Univariable case-control comparisons in cardiometabolic biomarkers^1^ at age 44-45 excluding cases endorsing only antipsychotic medication-related screening items (N = 26 excluded)

|  | **Psychosis Spectrum (N=145)** | | **Control**  **(N=2448)** | |  |  |
| --- | --- | --- | --- | --- | --- | --- |
|  |  | | | |  |  |
|  |  | | | |  |  |
| **Biomarker** | **mean** | **s.d.** | **mean** | **s.d.** | **t** | ***P*** |
|  | mg/dL or mg/L ^2^ | | | |  |  |
|  |  |  |  |  |  |  |
| Fibrinogen | 312.58 | 66.26 | 294.19 | 60.98 | -2.985 | **0.002** |
| C-reactive protein | 2.81 | 4.75 | 2.10 | 5.01 | -2.270 | **0.012** |
| High-density lipoprotein cholesterol | 56.88 | 14.74 | 60.94 | 15.41 | 2.656 | **0.004** |
| Low-density lipoprotein cholesterol | 133.04 | 34.71 | 132.01 | 34.62 | -0.287 | 0.387 |
|  |  | | | |  |  |
|  | DCCT-HbA1c ^3^ | | | |  |  |
|  |  |  |  |  |  |  |
| Glycated Haemoglobin (HbA1c) | 5.46 | 1.14 | 5.22 | 0.57 | -2.239 | **0.014** |
|  |  |  |  |  |  |  |
|  | **N** | **%** | **N** | **%** | **Pearson χ^2^** | ***P*** |
|  |  |  |  |  |  |  |
| Abdominal obesity | 65 | 45.8 | 665 | 27.2 | 22.73 | **<.001** |
| High blood pressure | 29 | 20.7 | 378 | 15.6 | 2.554 | 0.110 |
|  |  |  |  |  |  |  |
|  |  |  |  |  |  |  |

^1^ Descriptive characteristics and univariable group comparisons are based on the unimputed dataset.

^2^ Fibrinogen, high-density lipoprotein cholesterol and low-density lipoprotein cholesterol are expressed in mg/dL; c-reactive protein is expressed in mg/L.

^3^ DCCT-HbA1c is expressed as % of overall haemoglobin and aligned to the assay used in the Diabetes Control and Complications Trial (DCCT).

**Table S8** Sensitivity Analysis: Univariable case-control comparisons in cardiometabolic biomarkers^1^ at age 44-45 excluding cases endorsing only psychotic condition-related screening items (N = 6 excluded)

|  | **Psychosis Spectrum (N=165)** | | **Control**  **(N=2448)** | |  |  |
| --- | --- | --- | --- | --- | --- | --- |
|  |  | | | |  |  |
|  |  | | | |  |  |
| **Biomarker** | **mean** | **s.d.** | **mean** | **s.d.** | **t** | ***P*** |
|  | mg/dL or mg/L ^2^ | | | |  |  |
|  |  |  |  |  |  |  |
| Fibrinogen mg/DL | 314.43 | 66.77 | 294.19 | 60.98 | -3.373 | **<.001** |
| C-reactive protein mg/L | 2.73 | 4.32 | 2.10 | 5.01 | -2.662 | **0.004** |
| High-density lipoprotein chol mg/DL | 57.26 | 16.47 | 60.94 | 15.41 | 2.922 | **0.002** |
| Low-density lipoprotein chol mg/DL | 134.75 | 33.33 | 132.01 | 34.62 | -1.067 | 0.143 |
|  |  | | | |  |  |
|  | DCCT-HbA1c ^3^ | | | |  |  |
|  |  |  |  |  |  |  |
| Glycated Haemoglobin (HbA1c) | 5.43 | 1.08 | 5.22 | 0.57 | -3.561 | **<.001** |
|  |  |  |  |  |  |  |
|  | **N** | **%** | **N** | **%** | **Pearson χ^2^** | ***P*** |
|  |  |  |  |  |  |  |
| Abdominal obesity | 71 | 44.1 | 665 | 27.2 | 21.16 | **<.001** |
| High blood pressure | 33 | 20.5 | 378 | 15.6 | 2.666 | 0.103 |
|  |  |  |  |  |  |  |
|  |  |  |  |  |  |  |

^1^ Descriptive characteristics and univariable group comparisons are based on the unimputed dataset.

^2^ Fibrinogen, high-density lipoprotein cholesterol and low-density lipoprotein cholesterol are expressed in mg/dL; c-reactive protein is expressed in mg/L.

^3^ DCCT-HbA1c is expressed as % of overall haemoglobin and aligned to the assay used in the Diabetes Control and Complications Trial (DCCT).

**Table S9** Association of psychosis spectrum with glycated haemoglobin at age 44-45 (imputed dataset, N=7923)

| **Glycated Haemoglobin (HbA1c)** | **Coefficient** | **SE** | **t** | **P>t** | **95% CI** | |
| --- | --- | --- | --- | --- | --- | --- |
| Psychosis spectrum | 0.32 | 0.11 | 2.84 | **0.008** | 0.09 | 0.55 |
| Sex | -0.14 | 0.02 | -7.66 | **0.000** | -0.17 | -0.10 |
| Nocturnal enuresis at age 7 | 0.00 | 0.03 | 0.12 | 0.90 | -0.06 | 0.06 |
| Mother's age last birthday (yrs) | 0.00 | 0.00 | -1.24 | 0.22 | -0.01 | 0.00 |
| Ever breastfed | 0.02 | 0.02 | 1.01 | 0.31 | -0.02 | 0.07 |
| Low Birth Weight | 0.01 | 0.04 | 0.3 | 0.76 | -0.07 | 0.10 |
| Mother smoked during pregnancy | 0.02 | 0.02 | 1.08 | 0.28 | -0.02 | 0.06 |
| Mother employed up to CM’s age 5 | 0.01 | 0.02 | 0.57 | 0.57 | -0.03 | 0.06 |
| Housing tenure | -0.02 | 0.02 | -0.97 | 0.33 | -0.06 | 0.02 |
| Housing difficulties | -0.02 | 0.04 | -0.5 | 0.62 | -0.10 | 0.06 |
| Financial difficulties | -0.02 | 0.05 | -0.37 | 0.71 | -0.11 | 0.08 |
| Divorce/ separation by CM’s age 7 | 0.00 | 0.06 | -0.03 | 0.98 | -0.11 | 0.11 |
| Cognitive ability at age 7 | -0.06 | 0.01 | -4.01 | **0.000** | -0.08 | -0.03 |
| Externalising symptoms | 0.01 | 0.01 | 0.51 | 0.61 | -0.02 | 0.03 |
| Internalising symptoms | -0.10 | 0.04 | -2.54 | **0.01** | -0.18 | -0.02 |
| Summary of medical examinations at age 7 | 0.00 | 0.00 | -0.03 | 0.97 | -0.01 | 0.01 |
| Maternal separation for over 1 month | 0.00 | 0.03 | 0 | 1.00 | -0.07 | 0.07 |
| Parents want child to stay at school | 0.10 | 0.08 | 1.3 | 0.20 | -0.05 | 0.25 |
| Mother hardly ever reads to child | 0.02 | 0.03 | 0.66 | 0.51 | -0.04 | 0.08 |
| Mother not interested in child’s education | 0.03 | 0.03 | 0.89 | 0.37 | -0.03 | 0.09 |
| Number of household amenities | -0.02 | 0.01 | -1.05 | 0.29 | -0.04 | 0.01 |
| Mother not married at birth | 0.10 | 0.06 | 1.76 | 0.08 | -0.01 | 0.22 |
| Father’s social class at birth |  |  |  |  |  |  |
| *II* | 0.01 | 0.05 | 0.21 | 0.83 | -0.09 | 0.11 |
| *III* | 0.01 | 0.05 | 0.21 | 0.83 | -0.08 | 0.10 |
| *IV* | 0.08 | 0.06 | 1.42 | 0.16 | -0.03 | 0.19 |
| *V* | 0.06 | 0.06 | 1.06 | 0.29 | -0.05 | 0.18 |
| Region at CM’s birth |  |  |  |  |  |  |
| *North West* | 0.08 | 0.04 | 1.91 | 0.06 | 0.00 | 0.16 |
| *E & W. Riding* | 0.06 | 0.05 | 1.26 | 0.21 | -0.03 | 0.15 |
| *North Midlands* | 0.03 | 0.05 | 0.74 | 0.46 | -0.06 | 0.13 |
| *Midlands* | 0.05 | 0.05 | 1.21 | 0.23 | -0.03 | 0.14 |
| *East* | 0.06 | 0.05 | 1.17 | 0.24 | -0.04 | 0.15 |
| *South East* | 0.04 | 0.04 | 1.02 | 0.31 | -0.04 | 0.12 |
| *South* | 0.08 | 0.05 | 1.54 | 0.12 | -0.02 | 0.18 |
| *South West* | 0.05 | 0.05 | 0.96 | 0.34 | -0.05 | 0.15 |
| *Wales* | 0.05 | 0.05 | 0.93 | 0.36 | -0.05 | 0.15 |
| *Scotland* | 0.05 | 0.04 | 1.25 | 0.21 | -0.03 | 0.14 |
| Body mass index (BMI) at age 7 | 0.02 | 0.01 | 3.34 | **0.001** | 0.01 | 0.03 |
| _cons | 5.14 | 0.15 | 33.96 | 0.000 | 4.84 | 5.44 |

Grey shading indicates statistically significant predictors.

**Table S10** Association of psychosis spectrum with high-density lipoprotein (HDL) cholesterol at age 44-45 (imputed dataset, N=7808)

| **HDL** | | **Coefficient** | **SE** | **t** | **P>t** | **95% CI** | |
| --- | --- | --- | --- | --- | --- | --- | --- |
| Psychosis spectrum | | -4.47 | 1.65 | -2.71 | **0.009** | -7.78 | -1.16 |
| Sex | | 16.19 | 0.53 | 30.39 | **0.000** | 15.15 | 17.23 |
| Nocturnal enuresis at age 7 | | -1.20 | 0.85 | -1.41 | 0.16 | -2.87 | 0.46 |
| Mother's age last birthday (yrs) | | 0.03 | 0.05 | 0.65 | 0.52 | -0.07 | 0.13 |
| Ever breastfed | | -0.06 | 0.66 | -0.09 | 0.93 | -1.35 | 1.23 |
| Low Birth Weight | | 0.33 | 1.22 | 0.27 | 0.78 | -2.05 | 2.72 |
| Mother smoked during pregnancy | | 0.34 | 0.62 | 0.55 | 0.58 | -0.88 | 1.56 |
| Mother employed up to CM’s age 5 | | -0.83 | 0.64 | -1.31 | 0.19 | -2.08 | 0.42 |
| Housing tenure | | 2.56 | 0.64 | 4.02 | **0.000** | 1.31 | 3.82 |
| Housing difficulties | | 0.32 | 1.33 | 0.24 | 0.81 | -2.29 | 2.94 |
| Financial difficulties | | -0.50 | 1.40 | -0.35 | 0.72 | -3.26 | 2.26 |
| Divorce/ separation by CM’s age 7 | | -2.10 | 1.67 | -1.25 | 0.21 | -5.38 | 1.19 |
| Cognitive ability at age 7 | | 2.40 | 0.38 | 6.33 | **0.000** | 1.66 | 3.15 |
| Externalising symptoms | | -0.47 | 0.35 | -1.35 | 0.18 | -1.16 | 0.22 |
| Internalising symptoms | | -0.10 | 1.14 | -0.09 | 0.93 | -2.34 | 2.14 |
| Summary of medical examinations at age 7 | | -0.17 | 0.14 | -1.15 | 0.25 | -0.45 | 0.12 |
| Maternal separation for over 1 month | | -0.95 | 0.97 | -0.97 | 0.33 | -2.86 | 0.96 |
| Parents want child to stay at school | | 0.12 | 1.59 | 0.08 | 0.94 | -3.01 | 3.26 |
| Mother hardly ever reads to child | | -0.07 | 0.79 | -0.09 | 0.93 | -1.62 | 1.47 |
| Mother not interested in child’s education | | -0.74 | 0.94 | -0.79 | 0.43 | -2.58 | 1.11 |
| Number of household amenities | | 0.06 | 0.40 | 0.15 | 0.88 | -0.72 | 0.84 |
| Mother not married at birth | | -0.32 | 1.73 | -0.18 | 0.85 | -3.71 | 3.08 |
| Father’s social class at birth |  | |  |  |  |  |  |
| *II* | | -0.03 | 1.44 | -0.02 | 0.98 | -2.86 | 2.80 |
| *III* | | -2.58 | 1.32 | -1.96 | 0.05 | -5.16 | 0.00 |
| *IV* | | -5.17 | 1.53 | -3.38 | **0.001** | -8.17 | -2.17 |
| *V* | | -2.46 | 1.68 | -1.47 | 0.14 | -5.75 | 0.83 |
| Region at CM’s birth | |  |  |  |  |  |  |
| *North West* | | -2.48 | 1.26 | -1.96 | 0.05 | -4.96 | 0.00 |
| *E & W. Riding* | | -1.95 | 1.34 | -1.46 | 0.15 | -4.57 | 0.68 |
| *North Midlands* | | 0.14 | 1.37 | 0.1 | 0.92 | -2.55 | 2.82 |
| *Midlands* | | -0.05 | 1.32 | -0.03 | 0.97 | -2.64 | 2.54 |
| *East* | | -1.65 | 1.39 | -1.19 | 0.23 | -4.38 | 1.07 |
| *South East* | | -1.20 | 1.17 | -1.03 | 0.30 | -3.50 | 1.09 |
| *South* | | 0.48 | 1.47 | 0.33 | 0.75 | -2.41 | 3.37 |
| *South West* | | -2.58 | 1.49 | -1.73 | 0.08 | -5.50 | 0.34 |
| *Wales* | | -3.25 | 1.54 | -2.1 | **0.04** | -6.27 | -0.22 |
| *Scotland* | | -2.38 | 1.30 | -1.82 | 0.07 | -4.93 | 0.18 |
| Body mass index (BMI) at age 7 | | -0.76 | 0.17 | -4.55 | **0.000** | -1.09 | -0.43 |
| _cons | | 33.07 | 4.17 | 7.93 | 0.000 | 24.88 | 41.25 |

Grey shading indicates statistically significant predictors.

**Table S11** Association of psychosis spectrum with low-density lipoprotein (LDL) cholesterol at age 44-45 (imputed dataset, N=7391)

| **LDL Cholesterol** | **Coefficient** | **SE** | **t** | **P>t** | **95% CI** | |
| --- | --- | --- | --- | --- | --- | --- |
| Psychosis spectrum | 2.99 | 2.93 | 1.02 | 0.32 | -2.97 | 8.96 |
| Sex | -8.51 | 0.66 | -12.99 | **0.000** | -9.80 | -7.23 |
| Nocturnal enuresis at age 7 | -0.79 | 1.09 | -0.73 | 0.47 | -2.94 | 1.35 |
| Mother's age last birthday (yrs) | 0.01 | 0.06 | 0.2 | 0.84 | -0.11 | 0.13 |
| Ever breastfed | -0.57 | 0.82 | -0.7 | 0.49 | -2.18 | 1.04 |
| Low Birth Weight | -1.21 | 1.56 | -0.78 | 0.44 | -4.27 | 1.84 |
| Mother smoked during pregnancy | 0.50 | 0.74 | 0.68 | 0.49 | -0.94 | 1.95 |
| Mother employed up to CM’s age 5 | 1.08 | 0.85 | 1.27 | 0.21 | -0.59 | 2.75 |
| Housing tenure | -1.58 | 0.79 | -2 | **0.05** | -3.14 | -0.03 |
| Housing difficulties | -0.25 | 1.62 | -0.15 | 0.88 | -3.44 | 2.94 |
| Financial difficulties | -1.23 | 1.66 | -0.74 | 0.46 | -4.50 | 2.04 |
| Divorce/ separation by CM’s age 7 | -2.05 | 2.14 | -0.96 | 0.34 | -6.25 | 2.16 |
| Cognitive ability at age 7 | -0.29 | 0.48 | -0.61 | 0.54 | -1.24 | 0.65 |
| Externalising symptoms | 0.88 | 0.42 | 2.12 | **0.03** | 0.06 | 1.69 |
| Internalising symptoms | -1.30 | 1.43 | -0.91 | 0.36 | -4.10 | 1.50 |
| Summary of medical examinations at age 7 | 0.16 | 0.18 | 0.9 | 0.37 | -0.19 | 0.50 |
| Maternal separation for over 1 month | -2.51 | 1.20 | -2.1 | **0.04** | -4.86 | -0.16 |
| Parents want child to stay at school | 1.37 | 2.12 | 0.64 | 0.52 | -2.83 | 5.56 |
| Mother hardly ever reads to child | -2.27 | 0.99 | -2.3 | **0.02** | -4.21 | -0.33 |
| Mother not interested in child’s education | -0.39 | 1.22 | -0.32 | 0.75 | -2.79 | 2.01 |
| Number of household amenities | -0.01 | 0.50 | -0.03 | 0.98 | -1.00 | 0.97 |
| Mother not married at birth | -0.50 | 2.03 | -0.24 | 0.81 | -4.48 | 3.49 |
| Father’s social class at birth |  |  |  |  |  |  |
| *II* | -4.49 | 1.71 | -2.63 | **0.009** | -7.84 | -1.15 |
| *III* | -1.74 | 1.60 | -1.09 | 0.28 | -4.87 | 1.39 |
| *IV* | -1.42 | 1.89 | -0.75 | 0.45 | -5.13 | 2.29 |
| *V* | -1.58 | 2.00 | -0.79 | 0.43 | -5.51 | 2.35 |
| Region at CM’s birth |  |  |  |  |  |  |
| *North West* | -0.47 | 1.52 | -0.31 | 0.76 | -3.44 | 2.50 |
| *E & W. Riding* | 0.42 | 1.63 | 0.26 | 0.80 | -2.78 | 3.62 |
| *North Midlands* | -0.84 | 1.68 | -0.5 | 0.62 | -4.13 | 2.45 |
| *Midlands* | -0.23 | 1.59 | -0.14 | 0.89 | -3.35 | 2.89 |
| *East* | -2.17 | 1.70 | -1.27 | 0.20 | -5.51 | 1.17 |
| *South East* | -0.68 | 1.42 | -0.48 | 0.64 | -3.47 | 2.11 |
| *South* | -1.35 | 1.79 | -0.75 | 0.45 | -4.85 | 2.16 |
| *South West* | -0.86 | 1.87 | -0.46 | 0.65 | -4.53 | 2.80 |
| *Wales* | 0.85 | 1.90 | 0.45 | 0.65 | -2.87 | 4.57 |
| *Scotland* | -1.78 | 1.56 | -1.14 | 0.26 | -4.84 | 1.29 |
| Body mass index (BMI) at age 7 | -0.12 | 0.20 | -0.58 | 0.56 | -0.52 | 0.28 |
| _cons | 137.30 | 5.00 | 27.45 | 0.000 | 127.49 | 147.11 |

Grey shading indicates statistically significant predictors.

**Table S12** Association of psychosis spectrum with fibrinogen at age 44-45 (imputed dataset, N=7670)

| **Fibrinogen** | **Coefficient** | **SE** | **t** | **P>t** | **95% CI** | |
| --- | --- | --- | --- | --- | --- | --- |
| Psychosis spectrum | 4.54 | 1.78 | 2.55 | **0.02** | 0.94 | 8.14 |
| Sex | 5.07 | 0.46 | 10.98 | **0.000** | 4.16 | 5.97 |
| Nocturnal enuresis at age 7 | 0.40 | 0.74 | 0.54 | 0.59 | -1.06 | 1.86 |
| Mother's age last birthday (yrs) | -0.09 | 0.04 | -2.01 | 0.05 | -0.17 | 0.00 |
| Ever breastfed | -1.42 | 0.57 | -2.48 | **0.01** | -2.54 | -0.30 |
| Low Birth Weight | 0.18 | 1.04 | 0.17 | 0.86 | -1.86 | 2.23 |
| Mother smoked during pregnancy | 0.00 | 0.53 | -0.01 | 0.99 | -1.04 | 1.03 |
| Mother employed up to CM’s age 5 | -0.49 | 0.54 | -0.9 | 0.37 | -1.56 | 0.58 |
| Housing tenure | -1.95 | 0.54 | -3.64 | **0.000** | -3.00 | -0.90 |
| Housing difficulties | 0.07 | 1.14 | 0.06 | 0.95 | -2.17 | 2.31 |
| Financial difficulties | 0.74 | 1.22 | 0.61 | 0.54 | -1.66 | 3.15 |
| Divorce/ separation by CM’s age 7 | -0.15 | 1.38 | -0.11 | 0.91 | -2.86 | 2.56 |
| Cognitive ability at age 7 | -2.23 | 0.34 | -6.52 | **0.000** | -2.90 | -1.56 |
| Externalising symptoms | 1.02 | 0.29 | 3.49 | **0.000** | 0.45 | 1.59 |
| Internalising symptoms | -1.29 | 1.01 | -1.28 | 0.20 | -3.27 | 0.69 |
| Summary of medical examinations at age 7 | -0.02 | 0.12 | -0.13 | 0.90 | -0.26 | 0.23 |
| Maternal separation for over 1 month | 1.26 | 0.80 | 1.59 | 0.11 | -0.30 | 2.83 |
| Parents want child to stay at school | 0.60 | 1.44 | 0.41 | 0.68 | -2.25 | 3.44 |
| Mother hardly ever reads to child | 0.39 | 0.70 | 0.56 | 0.57 | -0.97 | 1.76 |
| Mother not interested in child’s education | 0.32 | 0.78 | 0.41 | 0.68 | -1.20 | 1.84 |
| Number of household amenities | 0.07 | 0.38 | 0.18 | 0.85 | -0.67 | 0.81 |
| Mother not married at birth | 0.60 | 1.50 | 0.4 | 0.69 | -2.35 | 3.55 |
| Father’s social class at birth |  |  |  |  |  |  |
| *II* | 0.65 | 1.23 | 0.53 | 0.60 | -1.77 | 3.07 |
| *III* | 3.05 | 1.13 | 2.7 | **0.007** | 0.84 | 5.26 |
| *IV* | 2.97 | 1.31 | 2.27 | **0.02** | 0.41 | 5.53 |
| *V* | 3.87 | 1.45 | 2.66 | **0.008** | 1.01 | 6.72 |
| Region at CM’s birth |  |  |  |  |  |  |
| *North West* | 0.94 | 1.08 | 0.87 | 0.39 | -1.18 | 3.05 |
| *E & W. Riding* | 0.01 | 1.16 | 0.01 | 0.99 | -2.26 | 2.29 |
| *North Midlands* | 0.12 | 1.19 | 0.1 | 0.92 | -2.21 | 2.46 |
| *Midlands* | 0.59 | 1.13 | 0.53 | 0.60 | -1.62 | 2.81 |
| *East* | 1.36 | 1.19 | 1.14 | 0.26 | -0.98 | 3.70 |
| *South East* | 1.77 | 1.01 | 1.75 | 0.08 | -0.21 | 3.76 |
| *South* | 2.72 | 1.28 | 2.12 | **0.03** | 0.21 | 5.23 |
| *South West* | 3.80 | 1.29 | 2.94 | **0.003** | 1.26 | 6.33 |
| *Wales* | 3.38 | 1.33 | 2.55 | **0.01** | 0.78 | 5.99 |
| *Scotland* | 0.58 | 1.11 | 0.52 | 0.60 | -1.60 | 2.76 |
| Body mass index (BMI) at age 7 | 0.87 | 0.15 | 5.62 | **0.000** | 0.56 | 1.17 |
| _cons | 84.75 | 3.69 | 22.99 | 0.000 | 77.51 | 91.99 |

Grey shading indicates statistically significant predictors.

**Table S13** Associations of psychosis spectrum with C-reactive protein (CRP) at age 44-45 (imputed dataset, N=7466)

| **C-Reactive Protein (CRP)** | **Coefficient** | **SE** | **t** | **P>t** | **95% CI** | |
| --- | --- | --- | --- | --- | --- | --- |
| Psychosis spectrum | 12.11 | 9.11 | 1.33 | 0.19 | -6.27 | 30.49 |
| Sex | 4.90 | 2.58 | 1.9 | 0.06 | -0.15 | 9.95 |
| Nocturnal enuresis at age 7 | 1.19 | 4.13 | 0.29 | 0.77 | -6.91 | 9.30 |
| Mother's age last birthday (yrs) | -0.40 | 0.24 | -1.67 | 0.10 | -0.87 | 0.07 |
| Ever breastfed | -1.77 | 3.12 | -0.57 | 0.57 | -7.89 | 4.35 |
| Low Birth Weight | -0.50 | 5.69 | -0.09 | 0.93 | -11.66 | 10.65 |
| Mother smoked during pregnancy | -1.27 | 2.84 | -0.45 | 0.65 | -6.84 | 4.29 |
| Mother employed up to CM’s age 5 | 4.03 | 3.02 | 1.34 | 0.18 | -1.89 | 9.95 |
| Housing tenure | -11.16 | 3.06 | -3.65 | **0.000** | -17.16 | -5.15 |
| Housing difficulties | -0.71 | 6.04 | -0.12 | 0.91 | -12.57 | 11.15 |
| Financial difficulties | -3.54 | 6.86 | -0.52 | 0.61 | -17.04 | 9.95 |
| Divorce/ separation by CM’s age 7 | 9.98 | 7.62 | 1.31 | 0.19 | -4.97 | 24.92 |
| Cognitive ability at age 7 | -12.86 | 1.85 | -6.93 | **0.000** | -16.49 | -9.22 |
| Externalising symptoms | 5.47 | 1.60 | 3.41 | **0.001** | 2.33 | 8.61 |
| Internalising symptoms | -8.46 | 5.52 | -1.53 | 0.13 | -19.29 | 2.37 |
| Summary of medical examinations at age 7 | 1.08 | 0.67 | 1.61 | 0.11 | -0.23 | 2.40 |
| Maternal separation for over 1 month | 4.63 | 4.47 | 1.03 | 0.30 | -4.14 | 13.40 |
| Parents want child to stay at school | 0.35 | 7.70 | 0.05 | 0.96 | -14.83 | 15.52 |
| Mother hardly ever reads to child | -1.29 | 3.90 | -0.33 | 0.74 | -8.95 | 6.38 |
| Mother not interested in child’s education | 3.46 | 4.36 | 0.79 | 0.43 | -5.09 | 12.01 |
| Number of household amenities | 0.61 | 2.12 | 0.29 | 0.78 | -3.56 | 4.77 |
| Mother not married at birth | -3.48 | 8.47 | -0.41 | 0.68 | -20.12 | 13.16 |
| Father’s social class at birth |  |  |  |  |  |  |
| *II* | -4.23 | 6.91 | -0.61 | 0.54 | -17.78 | 9.32 |
| *III* | 16.61 | 6.33 | 2.63 | **0.009** | 4.20 | 29.02 |
| *IV* | 20.12 | 7.28 | 2.76 | **0.006** | 5.84 | 34.39 |
| *V* | 16.05 | 8.17 | 1.96 | **0.05** | 0.01 | 32.08 |
| Region at CM’s birth |  |  |  |  |  |  |
| *North West* | -0.50 | 5.97 | -0.08 | 0.93 | -12.22 | 11.21 |
| *E & W. Riding* | 4.81 | 6.50 | 0.74 | 0.46 | -7.93 | 17.55 |
| *North Midlands* | -9.52 | 6.58 | -1.45 | 0.15 | -22.41 | 3.38 |
| *Midlands* | 2.08 | 6.35 | 0.33 | 0.74 | -10.36 | 14.53 |
| *East* | -7.40 | 6.66 | -1.11 | 0.27 | -20.46 | 5.67 |
| *South East* | 3.22 | 5.65 | 0.57 | 0.57 | -7.86 | 14.29 |
| *South* | 3.36 | 7.18 | 0.47 | 0.64 | -10.71 | 17.43 |
| *South West* | 0.79 | 7.28 | 0.11 | 0.91 | -13.50 | 15.07 |
| *Wales* | 17.52 | 7.37 | 2.38 | **0.02** | 3.08 | 31.97 |
| *Scotland* | 3.39 | 6.11 | 0.55 | 0.58 | -8.60 | 15.37 |
| Body mass index (BMI) at age 7 | 5.18 | 0.80 | 6.43 | **0.000** | 3.60 | 6.76 |
| _cons | -105.67 | 20.19 | -5.23 | 0.000 | -145.3 | -66.05 |

Grey shading indicates statistically significant predictors.

**Table S14** Association of psychosis spectrum with abdominal obesity at age 44-45 (imputed dataset, N=9298)

| **Abdominal Obesity** | **exp(b)** | **SE** | **t** | **P>t** | **95% CI** | |
| --- | --- | --- | --- | --- | --- | --- |
| Psychosis spectrum | 1.40 | 0.12 | 3.88 | **0.000** | 1.18 | 1.68 |
| Sex | 0.76 | 0.03 | -8.22 | **0.000** | 0.72 | 0.81 |
| Nocturnal enuresis at age 7 | 1.10 | 0.05 | 2.11 | **0.04** | 1.01 | 1.20 |
| Mother's age last birthday (yrs) | 1.00 | 0.00 | -1.51 | 0.13 | 0.99 | 1.00 |
| Ever breastfed | 1.00 | 0.04 | -0.09 | 0.93 | 0.93 | 1.07 |
| Low Birth Weight | 0.87 | 0.07 | -1.77 | 0.08 | 0.75 | 1.02 |
| Mother smoked during pregnancy | 1.10 | 0.04 | 2.86 | **0.004** | 1.03 | 1.18 |
| Mother employed up to CM’s age 5 | 0.98 | 0.04 | -0.61 | 0.54 | 0.91 | 1.05 |
| Housing tenure | 0.88 | 0.03 | -3.18 | **0.002** | 0.82 | 0.95 |
| Housing difficulties | 0.95 | 0.07 | -0.69 | 0.49 | 0.83 | 1.09 |
| Financial difficulties | 0.94 | 0.07 | -0.76 | 0.45 | 0.81 | 1.10 |
| Divorce/ separation by CM’s age 7 | 0.95 | 0.09 | -0.5 | 0.61 | 0.79 | 1.15 |
| Cognitive ability at age 7 | 0.83 | 0.02 | -8.83 | **0.000** | 0.79 | 0.86 |
| Externalising symptoms | 1.05 | 0.02 | 2.84 | **0.005** | 1.02 | 1.09 |
| Internalising symptoms | 0.89 | 0.09 | -1.14 | 0.25 | 0.74 | 1.08 |
| Summary of medical examinations at age 7 | 1.01 | 0.01 | 0.72 | 0.47 | 0.99 | 1.02 |
| Maternal separation for over 1 month | 1.04 | 0.06 | 0.77 | 0.44 | 0.94 | 1.16 |
| Parents want child to stay at school | 0.97 | 0.08 | -0.35 | 0.73 | 0.82 | 1.14 |
| Mother hardly ever reads to child | 1.06 | 0.05 | 1.26 | 0.21 | 0.97 | 1.15 |
| Mother not interested in child’s education | 1.00 | 0.05 | -0.09 | 0.93 | 0.91 | 1.09 |
| Number of household amenities | 0.98 | 0.02 | -0.98 | 0.33 | 0.93 | 1.02 |
| Mother not married at birth | 0.89 | 0.10 | -1.01 | 0.31 | 0.72 | 1.11 |
| Father’s social class at birth |  |  |  |  |  |  |
| *II* | 1.09 | 0.12 | 0.78 | 0.43 | 0.88 | 1.35 |
| *III* | 1.28 | 0.13 | 2.48 | **0.01** | 1.05 | 1.56 |
| *IV* | 1.30 | 0.14 | 2.39 | **0.02** | 1.05 | 1.61 |
| *V* | 1.28 | 0.15 | 2.16 | **0.03** | 1.02 | 1.60 |
| Region at CM’s birth |  |  |  |  |  |  |
| *North West* | 1.17 | 0.09 | 2.09 | **0.04** | 1.01 | 1.35 |
| *E & W. Riding* | 0.92 | 0.08 | -1 | 0.32 | 0.77 | 1.09 |
| *North Midlands* | 0.91 | 0.08 | -1.08 | 0.28 | 0.77 | 1.08 |
| *Midlands* | 1.13 | 0.09 | 1.51 | 0.13 | 0.97 | 1.31 |
| *East* | 1.11 | 0.09 | 1.22 | 0.22 | 0.94 | 1.31 |
| *South East* | 1.07 | 0.08 | 0.96 | 0.34 | 0.93 | 1.23 |
| *South* | 0.92 | 0.09 | -0.85 | 0.39 | 0.76 | 1.11 |
| *South West* | 0.89 | 0.09 | -1.24 | 0.22 | 0.73 | 1.07 |
| *Wales* | 1.14 | 0.10 | 1.52 | 0.13 | 0.96 | 1.36 |
| *Scotland* | 1.02 | 0.08 | 0.27 | 0.79 | 0.88 | 1.19 |
| Body mass index (BMI) at age 7 | 1.09 | 0.01 | 8.83 | **0.000** | 1.07 | 1.11 |
| _cons | 0.10 | 0.03 | -9.07 | 0.000 | 0.06 | 0.17 |

Grey shading indicates statistically significant predictors.

**Table S15** Association of psychosis spectrum with high blood pressure at age 44-45 (imputed dataset, N=9229)

| **High Blood Pressure** | **exp(b)** | **SE** | **t** | **P>t** | **95% CI** | |
| --- | --- | --- | --- | --- | --- | --- |
| Psychosis spectrum | 1.20 | 0.20 | 1.09 | 0.28 | 0.86 | 1.69 |
| Sex | 0.53 | 0.03 | -12.21 | **0.000** | 0.48 | 0.59 |
| Nocturnal enuresis at age 7 | 1.01 | 0.07 | 0.11 | 0.92 | 0.87 | 1.17 |
| Mother's age last birthday (yrs) | 1.00 | 0.00 | -1.07 | 0.28 | 0.99 | 1.00 |
| Ever breastfed | 1.14 | 0.07 | 2.27 | **0.02** | 1.02 | 1.28 |
| Low Birth Weight | 1.34 | 0.13 | 2.99 | **0.003** | 1.11 | 1.62 |
| Mother smoked during pregnancy | 1.02 | 0.06 | 0.33 | 0.74 | 0.92 | 1.13 |
| Mother employed up to CM’s age 5 | 1.02 | 0.06 | 0.3 | 0.77 | 0.91 | 1.13 |
| Housing tenure | 0.90 | 0.05 | -1.8 | 0.07 | 0.80 | 1.01 |
| Housing difficulties | 0.92 | 0.10 | -0.74 | 0.46 | 0.73 | 1.15 |
| Financial difficulties | 0.95 | 0.12 | -0.42 | 0.68 | 0.74 | 1.22 |
| Divorce/ separation by CM’s age 7 | 0.88 | 0.13 | -0.82 | 0.41 | 0.65 | 1.19 |
| Cognitive ability at age 7 | 0.94 | 0.03 | -2.01 | **0.04** | 0.88 | 1.00 |
| Externalising symptoms | 0.95 | 0.03 | -1.61 | 0.11 | 0.90 | 1.01 |
| Internalising symptoms | 1.12 | 0.12 | 1.1 | 0.27 | 0.91 | 1.38 |
| Medical examination summary at age 7 | 1.03 | 0.01 | 2.08 | **0.04** | 1.00 | 1.05 |
| Maternal separation for over 1 month | 0.98 | 0.08 | -0.2 | 0.84 | 0.83 | 1.16 |
| Parents want child to stay at school | 1.02 | 0.15 | 0.13 | 0.90 | 0.76 | 1.38 |
| Mother hardly ever reads to child | 0.96 | 0.07 | -0.62 | 0.54 | 0.83 | 1.10 |
| Mother not interested in child’s education | 0.98 | 0.08 | -0.29 | 0.77 | 0.84 | 1.14 |
| Number of household amenities | 0.95 | 0.03 | -1.46 | 0.14 | 0.88 | 1.02 |
| Mother not married at birth | 1.04 | 0.17 | 0.27 | 0.79 | 0.76 | 1.42 |
| Father’s social class at birth |  |  |  |  |  |  |
| *II* | 1.33 | 0.21 | 1.81 | 0.07 | 0.98 | 1.81 |
| *III* | 1.39 | 0.20 | 2.22 | **0.03** | 1.04 | 1.85 |
| *IV* | 1.55 | 0.25 | 2.72 | **0.007** | 1.13 | 2.14 |
| *V* | 1.68 | 0.28 | 3.09 | **0.002** | 1.21 | 2.35 |
| Region at CM’s birth |  |  |  |  |  |  |
| *North West* | 0.97 | 0.12 | -0.23 | 0.82 | 0.77 | 1.23 |
| *E & W. Riding* | 1.16 | 0.14 | 1.18 | 0.24 | 0.91 | 1.48 |
| *North Midlands* | 1.07 | 0.13 | 0.5 | 0.62 | 0.83 | 1.36 |
| *Midlands* | 1.18 | 0.14 | 1.39 | 0.17 | 0.94 | 1.48 |
| *East* | 0.91 | 0.12 | -0.67 | 0.50 | 0.70 | 1.19 |
| *South East* | 1.07 | 0.12 | 0.59 | 0.55 | 0.86 | 1.32 |
| *South* | 1.03 | 0.14 | 0.18 | 0.86 | 0.78 | 1.35 |
| *South West* | 0.85 | 0.13 | -1.08 | 0.28 | 0.64 | 1.14 |
| *Wales* | 1.24 | 0.17 | 1.57 | 0.12 | 0.95 | 1.61 |
| *Scotland* | 1.06 | 0.13 | 0.51 | 0.61 | 0.84 | 1.35 |
| Body mass index (BMI) at age 7 | 1.04 | 0.02 | 2.7 | **0.007** | 1.01 | 1.07 |
| _cons | 0.14 | 0.05 | -5.15 | 0.000 | 0.07 | 0.30 |

Grey shading indicates statistically significant predictors.

**Table S16** Prevalence of pre-diabetes, diabetes and metabolic syndrome at age 44-45 in participants on the psychosis spectrum (N=171) and controls (N=2448) (unimputed dataset)

| **Variables** |  | **Psychosis spectrum**  **(n=171)** | **Controls**  **(n=2448)** |
| --- | --- | --- | --- |
|  | **Abnormal Range** | **N (%)** | **N (%)** |
| Glycated Haemoglobin (HbA1c), DCCT-% | Prediabetes (5.7-6.4) | 17 (13.3) | 148 (7.19) |
|  | Diabetes (>6.4) | 4 (3.1) | 39 (1.9) |
| Metabolic risk profile | Metabolic Syndrome ^a^ | 42 (24.6) | 415 (17) |

DCCT: Diabetes Control and Complications Trial
^a^ For the definition of metabolic syndrome in our study, see Appendix 1 in this Supplement.

**Figure S1** Analytic cohort derivation (unimputed dataset)

**
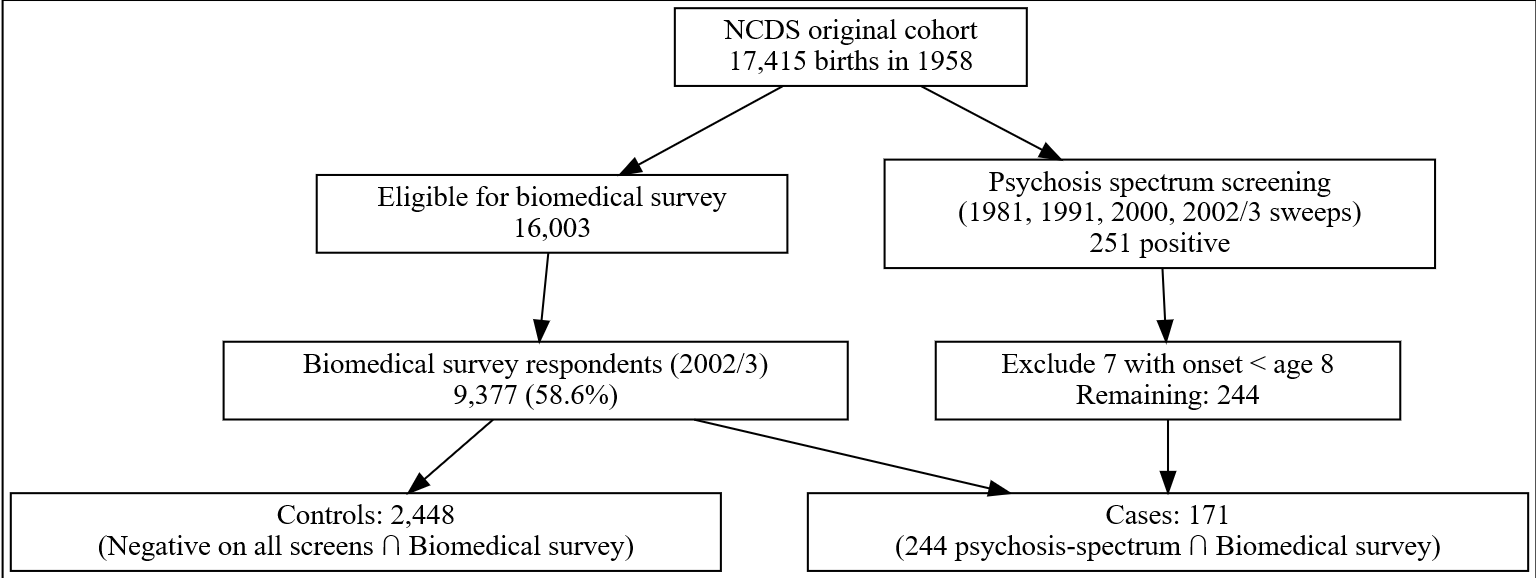
**

**Figure S2** Directed acyclic graph illustrating the hypothesised prospective association^1^ between Psychosis-Spectrum Status and Cardiometabolic Outcomes at Age 44-45

Early-Life Adversity at Ages 0-7 ───────────────────────────▶ Cardiometabolic Outcomes at Age 44-45

│

▼

Psychosis-Spectrum Status in Adulthood (ages 23 to 44-45)

│

▼

Adult Behaviours & Treatment Factors

(e.g. Smoking, Physical Activity, Diet, Antipsychotics)

│

▼

Cardiometabolic Outcomes at Age 44–45

^1^ Early-life adversity (0-7 years) is conceptualised as a confounder of the prospective association between psychosis-spectrum status and cardiometabolic outcomes at age 44-45. Psychoses-related factors (e.g. adult behavioural factors, antipsychotic exposure) are treated as mediators on the pathway from psychosis-spectrum status to cardiometabolic biomarkers at age 44-45. Multivariable models adjust for early-life adversity to estimate the total effect of psychosis-spectrum status on cardiometabolic outcomes.

**Figure S3** Associations of Psychosis Spectrum with Continuous Cardiometabolic Biomarkers at Age 44-45 (Percentage Differences in Means between Cases and Controls)


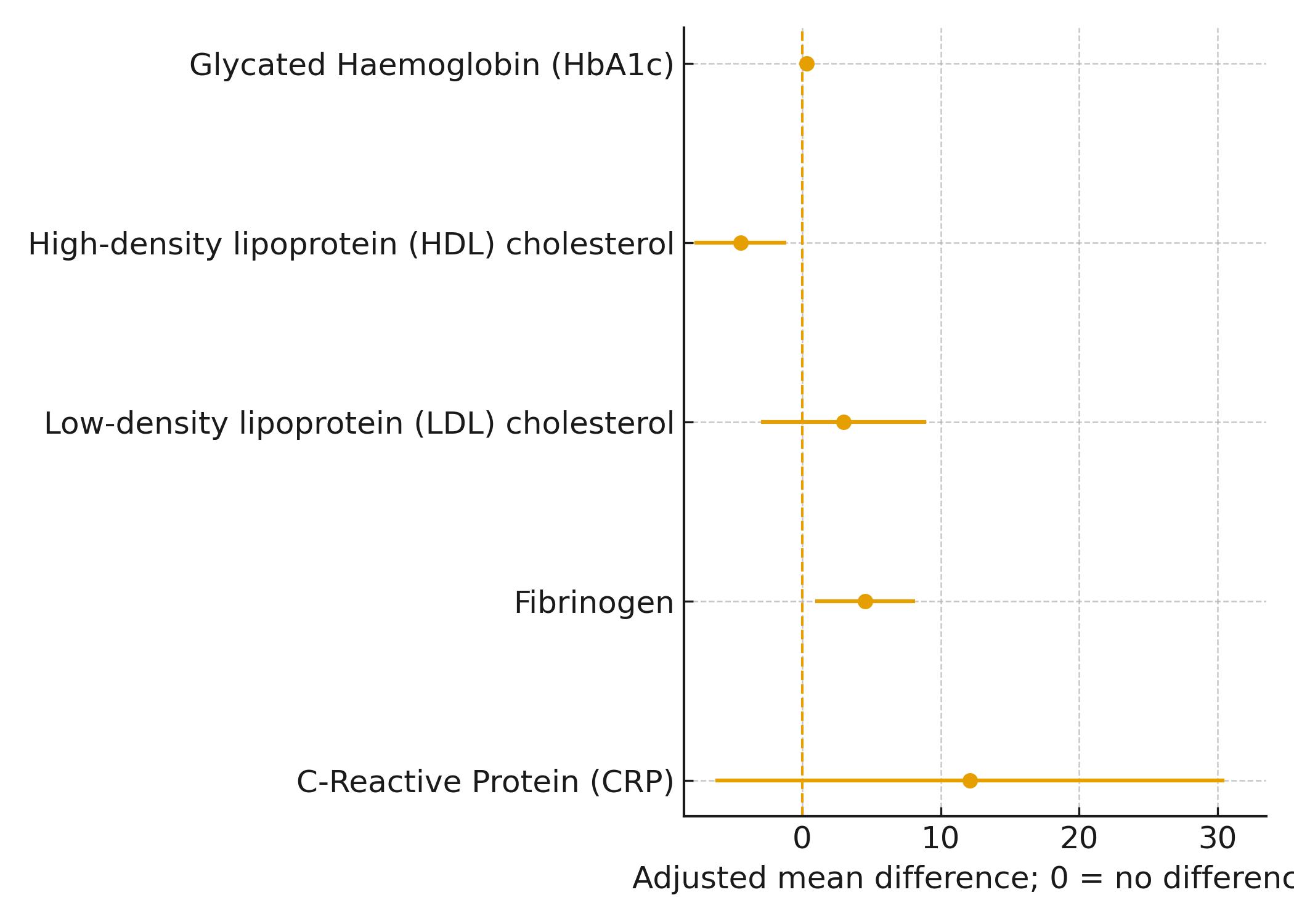


Notes: Points show percentage differences in means between cases and controls; horizontal lines show 95% confidence intervals. The vertical line at 0 denotes no percentage difference between groups.

**Figure S4** Associations of Psychosis Spectrum with Binary Cardiometabolic Outcomes at Age 44-45 (Odds Ratios)

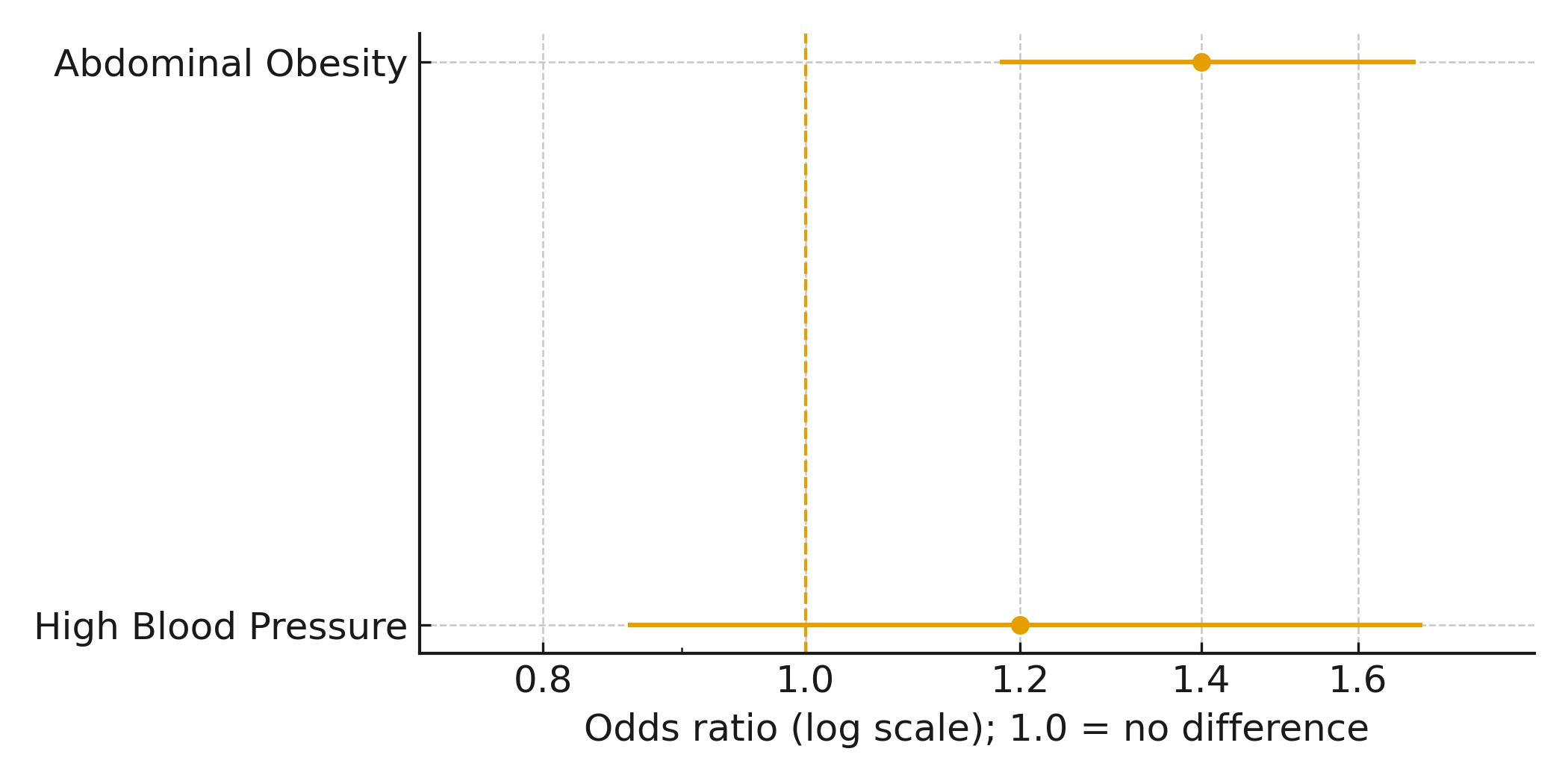


Notes: Points show odds ratio estimates; horizontal lines show 95% confidence intervals. The x-axis is on a logarithmic scale so that confidence intervals are symmetric around 1.0 (no difference). Values to the right of 1.0 indicate higher odds in the psychosis-spectrum group, and values to the left indicate lower odds. For example, 1.20 corresponds to a 20% higher odds and 0.80 would correspond to 20% lower odds.

**Appendix 1** Metabolic Syndrome

We adapted the ATP (Adult Treatment Panel) III-A Definition,^1^ replacing the fasting plasma glucose (FPG) criterion with a HbA1c cutoff of 5.4%. The latter criterion has been validated in Caucasian populations^1^

**Table A1** Adult Treatment Protocol- Adapted (ATP-III-A) definition of the metabolic syndrome^2^

| **Measure** | **Criteria (3/5 required)** |
| --- | --- |
| Waist (cm) | Males > 102, Females > 88 |
| Blood pressure (mmHg) | ≥130/85* |
| HDL (mg/dl) | Males < 40, Females < 50 |
| Triglycerides (mg/dl) | ≥150 |
| Glucose (mg/dl)** | ≥100 |

* Or treated with antihypertensive medication

** For the present study, the fasting plasma glucose (FPG) criterion was replaced by a HbA1c cutoff of 5.4%, which has been validated in Caucasian populations.^1^

**Appendix 2** Indicators of Early-Life Adversity (ELA)

**Birth characteristics:** birthweight; maternal smoking during pregnancy; maternal age,

maternal partnerships status, whether the CMs were ever breastfed, region of

residence at CM’s birth.

**Parental characteristics:** maternal employment up to CM’s age 5, mother reading to child,

parental interest in school, divorce by CM’s age 7, and separation from child from more than one month.

**Indicators of early life socio-economic position**: paternal social class at birth; financial

difficulties at CM’s age 7; housing tenure at CM’s age 7; access to household amenities* and housing difficulties at age 7.

**Characteristics of the CM at age 7**: cognitive ability**, internalising and externalising symptoms, nocturnal enuresis, a summary of health conditions assessed in the medical visit at age 7***, and the Body Mass Index (BMI).

** Access to household amenities*: Sum of sole use of: indoor toilet, outside toilet, cooking

facilities, hot water supply, garden or yard

***Cognitive ability at 7*: Principal Component Analysis (PCA) score. PCA indicators were the

Problem Arithmetic Test score, Total score on Copying Designs Test, Drawing a Man Test

score and the Southgate Group Reading Test score.

****Sum score of health/medical conditions at 7:* Measles/German measles, whooping

cough, chicken pox, mumps, scarlet fever, glandular fever, heart conditions, disability,

hernia, spasms, limb defect, malfunction of upper limb, stuttering, skull deformity, spinal

disorders, congenital lower limb defect, talipes, malfunction of the lower limb, neurological

skeletal disorder, asthma, mouth or palate abnormality, major handicap/disfiguring condition, abnormal chest shape, eczema, external ear deformity.

**Appendix 3** Missing Data

Missing data due to non-response are unavoidable in longitudinal surveys and may result in reduced sample size, diminished statistical power, and decreased representativeness relative to the originally intended target population. Rubin^3-5^ described three missing data mechanisms: Missing Completely at Random (MCAR), Missing at Random (MAR), and Missing Not at Random (MNAR).^3-5^ MCAR implies no systematic differences between observed and missing data, whereas MAR assumes that systematic differences can be explained by observed variables such that, conditional on observed data, missingness does not depend on unobserved values. With limited exceptions for specific missing data patterns,^6,7^ the MAR assumption is not directly testable.^8^ MNAR implies that observed data are insufficient to explain missingness and typically requires strong additional assumptions for valid inference.^9^

Within the context of the National Child Development Study, the missing data mechanism is unlikely to be MCAR, as previous research has demonstrated systematic associations between participant characteristics and non-response.^10-12^ In practice, as is typical for longitudinal cohort studies, missingness in the NCDS is most plausibly MAR, though MNAR cannot be excluded.^8^

We addressed missing data using multiple imputation (MI) by chained equations, generating 25 imputed datasets.^13^ MI operates under the MAR assumption,^3^ such that valid inference depends on the inclusion of variables predictive of both missingness and study outcomes.^14,15^ Following established recommendations, the imputation models incorporated all exposures, potential confounders, cardiometabolic outcomes, and a range of auxiliary variables selected for their strong associations with both biomedical risk factors and non-response in the biomedical sweep.^12,14^ This approach aimed to enhance the plausibility of the MAR assumption and to restore sample representativeness.

Imputation was conducted for participants who were alive and residing in Britain at the time of the 2002/03 biomedical sweep, reflecting the target population for the analyses. As missingness occurred in exposures, confounders, and outcomes, cardiometabolic biomarkers were included in the imputation models. Consistent with recommendations by von Hippel,^16^ imputed outcome values were not retained for primary analyses; however, analyses retaining imputed biomarker values yielded materially similar results (available upon request).

All statistical models were estimated separately within each imputed dataset, and parameter estimates were subsequently pooled using Rubin’s rules^3,5^ and standard multiple imputation combining procedures as described by Enders^17^ to obtain combined point estimates and robust standard errors that appropriately account for within- and between-imputation variability.

**Note:** While the distinction between MAR and MNAR mechanisms is not directly testable, extensive methodological work in the NCDS has demonstrated that leveraging rich pre-attrition data within multiple imputation substantially reduces selection bias and restores representativeness to both internal and external benchmarks.^12^ In contrast, complete-case analysis would rely on the implausible assumption of missing completely at random, which has been shown not to hold in this cohort.^18^ Given that missingness affected exposures, outcomes, and confounders, MI-based estimates were therefore prioritised as the primary inferential results, consistent with best practice for longitudinal cohort studies.

**Appendix 4** STROBE Statement—checklist of items that should be included in reports of observational studies

|  | **Item No.** | **Recommendation** | **Page  No.** |  |
| --- | --- | --- | --- | --- |
| **Title and abstract** | 1 | (*a*) Indicate the study’s design with a commonly used term in the title or the abstract | 1-3 | |
|  |  | (*b*) Provide in the abstract an informative and balanced summary of what was done and what was found | 2-3 | |
| **Introduction** | | | | |
| Background/rationale | 2 | Explain the scientific background and rationale for the investigation being reported | 4-6 | |
| Objectives | 3 | State specific objectives, including any prespecified hypotheses | 5-6 | |
| **Methods** | | | | |
| Study design | 4 | Present key elements of study design early in the paper | 6 | |
| Setting | 5 | Describe the setting, locations, and relevant dates, including periods of recruitment, exposure, follow-up, and data collection | 6-12 | |
| Variables | 7 | Clearly define all outcomes, exposures, predictors, potential confounders, and effect modifiers. Give diagnostic criteria, if applicable | 6-11 | |
| Data sources/ measurement | 8* | For each variable of interest, give sources of data and details of methods of assessment (measurement). Describe comparability of assessment methods if there is more than one group | 8-12  Supplementary Table S1 | |
| Bias | 9 | Describe any efforts to address potential sources of bias | 10-12 | |
| Study size | 10 | Explain how the study size was arrived at | 5-9  Supplementary Table S1 | |

| Quantitative variables | 11 | Explain how quantitative variables were handled in the analyses. If applicable, describe which groupings were chosen and why | - |
| --- | --- | --- | --- |
| Statistical methods | 12 | (*a*) Describe all statistical methods, including those used to control for confounding | 10-12 |
|  |  | (*b*) Describe any methods used to examine subgroups and interactions | 11 |
|  |  | (*c*) Explain how missing data were addressed | 10  Appendix 3 (Supplementary Material) |
|  |  | (*d*) *Cohort study*—If applicable, explain how loss to follow-up was addressed | 6-7, 10 |
|  |  | (*e*) Describe any sensitivity analyses | 11 |
| **Results** |  |  |  |
| Participants | 13* | (a) Report numbers of individuals at each stage of study—eg numbers potentially eligible, examined for eligibility, confirmed eligible, included in the study, completing follow-up, and analysed | (6-10)  27-28 (Tables 2, 3)  Supplementary Tables S1-S16 |
|  |  | (b) Give reasons for non-participation at each stage | (6-9) |
|  |  | (c) Consider use of a flow diagram | - |
| Descriptive data | 14* | (a) Give characteristics of study participants (eg demographic, clinical, social) and information on exposures and potential confounders | (6-10), Table 2  Supplementary Tables S2-S3 |
|  |  | (b) Indicate number of participants with missing data for each variable of interest | 28 (Table 3)  Supplementary Tables S1, S3, S9-S15 |
|  |  | (c) *Cohort study*—Summarise follow-up time (eg, average and total amount) | (6) |
| Outcome data | 15* | *Cohort study*—Report numbers of outcome events or summary measures over time | 26-28 (Tables 1-3)  Supplementary Tables S9-S16 |
| Main results | 16 | (*a*) Give unadjusted estimates and, if applicable, confounder-adjusted estimates and their precision (eg, 95% confidence interval). Make clear which confounders were adjusted for and why they were included | 26, 28 (Tables 1, 3)  Supplementary Tables S9-15 |
|  |  | (*b*) Report category boundaries when continuous variables were categorized | Appendix 2, Supplementary Material, Supplementary Table S16 |
|  |  | (*c*) If relevant, consider translating estimates of relative risk into absolute risk for a meaningful time period | n/a |

| Other analyses | 17 | Report other analyses done—eg analyses of subgroups and interactions, and sensitivity analyses | 14  Supplementary Table S4-S8 |
| --- | --- | --- | --- |
| **Discussion** |  |  |  |
| Key results | 18 | Summarise key results with reference to study objectives | 14-16 |
| Limitations | 19 | Discuss limitations of the study, taking into account sources of potential bias or imprecision. Discuss both direction and magnitude of any potential bias | 18-19 |
| Interpretation | 20 | Give a cautious overall interpretation of results considering objectives, limitations, multiplicity of analyses, results from similar studies, and other relevant evidence | 14-19 |
| Generalisability | 21 | Discuss the generalisability (external validity) of the study results | 19 |
| **Other information** | |  |  |
| Funding | 22 | Give the source of funding and the role of the funders for the present study and, if applicable, for the original study on which the present article is based | 20-21 |

*Give information separately for cases and controls in case-control studies and, if applicable, for exposed and unexposed groups in cohort and cross-sectional studies.

**Note:** An Explanation and Elaboration article discusses each checklist item and gives methodological background and published examples of transparent reporting. The STROBE checklist is best used in conjunction with this article (freely available on the Web sites of PLoS Medicine at http://www.plosmedicine.org/, Annals of Internal Medicine at http://www.annals.org/, and Epidemiology at http://www.epidem.com/). Information on the STROBE Initiative is available at www.strobe-statement.org.

**References**

1. Veeranna V, Ramesh K, Zalawadiya SK, Niraj A, Pradhan J, Jacob S, Afonso L. Glycosylated hemoglobin and prevalent metabolic syndrome in nondiabetic multiethnic U.S. adults. *Metab Syndr Relat Disord* Oct 2011;9(5):361-367.

2. Grundy SM, Cleeman JI, Daniels SR, et al. Diagnosis and management of the metabolic syndrome: an American Heart Association/National Heart, Lung, and Blood Institute Scientific Statement. *Circulation* Oct 25 2005;112(17):2735-2752.

3. Little RJA, Rubin DB. *Statistical Analysis with Missing Data*. 2nd ed. Chichester: Wiley; 2002.

4. Hughes RA, Heron J, Sterne JAC, Tilling K. Accounting for missing data in statistical analyses: multiple imputation is not always the answer. *Int J Epidemiol* Aug 1 2019;48(4):1294-1304.

5. Little RJA, Rubin DB. The Analysis of Social Science Data with Missing Values. *Sociological Methods & Research* 1989/11/01 1989;18(2-3):292-326.

6. Mohan K, Pearl J, Tian J. Graphical models for inference with missing data. *Proceedings of the 27th International Conference on Neural Information Processing Systems – Volume 1.* Lake Tahoe, Nevada; 2013.

7. Robins JM, Gill RD. Non-response models for the analysis of non-monotone ignorable missing data. Stat Med Jan 15-Feb 15 1997;16(1-3):39-56.

8. Molenberghs G, Beunckens C, Sotto C, Kenward MG. Every missingness not at random model has a missingness at random counterpart with equal fit. *Journal of the Royal Statistical Society: Series B (Statistical Methodology)* 2008;70:371-388.

9. Muthen B, Asparouhov T, Hunter AM, Leuchter AF. Growth modeling with nonignorable dropout: alternative analyses of the STAR*D antidepressant trial. *Psychol Methods* Mar 2011;16(1):17-33.

10. Atherton K, Fuller E, Shepherd P, Strachan DP, Power C. Loss and representativeness in a biomedical survey at age 45 years: 1958 British birth cohort. *J Epidemiol Community Health* Mar 2008;62(3):216-223.

11. Hawkes D, Plewis I. Modelling non‐response in the National Child Development Study. *Journal of the Royal Statistical Society Series A* 2006;169(3):479-491.

12. Mostafa T, Narayanan M, Pongiglione B, Dodgeon B, Goodman A, Silverwood RJ, Ploubidis GB. *Improving the plausibility of the missing at random assumption in the 1958 British birth cohort: a pragmatic data-driven approach*. London: UCL Centre for Longitudinal Studies; 2020.

13. White IR, Royston P, Wood AM. Multiple imputation using chained equations: Issues and guidance for practice. *Stat Med* Feb 20 2011;30(4):377-399.

14. Carpenter J, Kenward M. Multiple Imputation And Its Application. 12/01 2012.

15. White IR, Royston P. Imputing missing covariate values for the Cox model. *Stat Med* Jul 10 2009;28(15):1982-1998.

16. von Hippel PT. Regression with missing Ys: An improved strategy for analyzing multiply imputed data. *Sociological Methodology* 2007;37:83-117.

17. Enders CK. *Applied Missing Data Analysis.* New York: Guilford Press; 2010.

18. Bartlett JW, Carpenter JR, Tilling K, Vansteelandt S. Improving upon the efficiency of complete case analysis when covariates are MNAR. *Biostatistics*. 2014 Oct;15(4):719-30. doi: 10.1093/biostatistics/kxu023.
